# Thoughts on the popularity of ICSI

**DOI:** 10.1007/s10815-020-01987-0

**Published:** 2020-11-06

**Authors:** Mounia Haddad, Joshua Stewart, Philip Xie, Stephanie Cheung, Aysha Trout, Derek Keating, Alessandra Parrella, Sherina Lawrence, Zev Rosenwaks, Gianpiero D. Palermo

**Affiliations:** The Ronald O. Perelman and Claudia Cohen Center for Reproductive Medicine, Weill Cornell Medicine, New York, NY USA

**Keywords:** ICSI, IVF, Infertility, ART, History of ICSI, Application of ICSI, Development of ICSI, Versatility of ICSI, Popularity of ICSI

## Abstract

**Purpose:**

Intracytoplasmic sperm injection (ICSI) is the most widely utilized assisted reproductive technique (ART) worldwide. In this feature, we review the early assisted fertilization attempts that eventually led to the development of ICSI, and discuss its current utilization in cases of male and non-male factor infertility.

**Methods:**

We researched the literature related to the development, indications, and current use of ICSI, such as sperm structural abnormalities, male genetic indications, surgically retrieved sperm, high sperm chromatin fragmentation, oocyte dysmorphism, and preimplantation genetic testing (PGT). We also describe the potential future applications of ICSI.

**Results:**

This review summarizes the early micromanipulation techniques that led to the inception of ICSI. We also explore its current indications, including non-male factor infertility, where its use is more controversial. Finally, we consider the benefits of future advancements in reproductive biology that may incorporate ICSI, such as in vitro spermatogenesis, neogametogenesis, and heritable genome editing.

**Conclusion:**

The versatility, consistency, and reliability of ICSI have made it the most prevalently utilized ART procedure worldwide.

## Introduction

The topic of infertility has been explored across a wide range of fields- medicine, social science, religion, and philosophy. Infertility has led to the appearance of what is known today as assisted reproductive technology (ART), with intracytoplasmic sperm injection (ICSI) becoming the most widely used insemination method worldwide. Indeed, ICSI is used today in 66.5% [1] of fertility centers. The reliability of ICSI to achieve fertilization in cases of severe male factor infertility has led to the dramatic expansion of its use; it is now commonly utilized for low oocyte maturity, with cryopreserved oocytes, in conjunction with preimplantation genetic testing (PGT) and for patients of advanced maternal age (AMA) [1].

In 2003, the Society for Assisted Reproductive Technology (SART) reported a total of 112,988 annual ART cycles in the United States, which steadily grew to 248,086 cycles by 2017 [2]. Along with this growth, patients are opting for additional ART procedures to enhance their chances of having a successful pregnancy, including oocyte cryopreservation to postpone conception, and PGT to assess the genetic profile of embryos and increase the likelihood of implantation [2]. As the overall incidence rate of cancer patients in young adults (age 15–39) in the United States is increasing by 0.9% each year [3], many patients are also choosing oocyte cryopreservation to preserve their fertility. All these factors have contributed to the growing popularity of ICSI. Yet despite its popularity, whether ICSI is actually more beneficial than standard in vitro insemination has been the subject of debate.

**Fig. 1:**
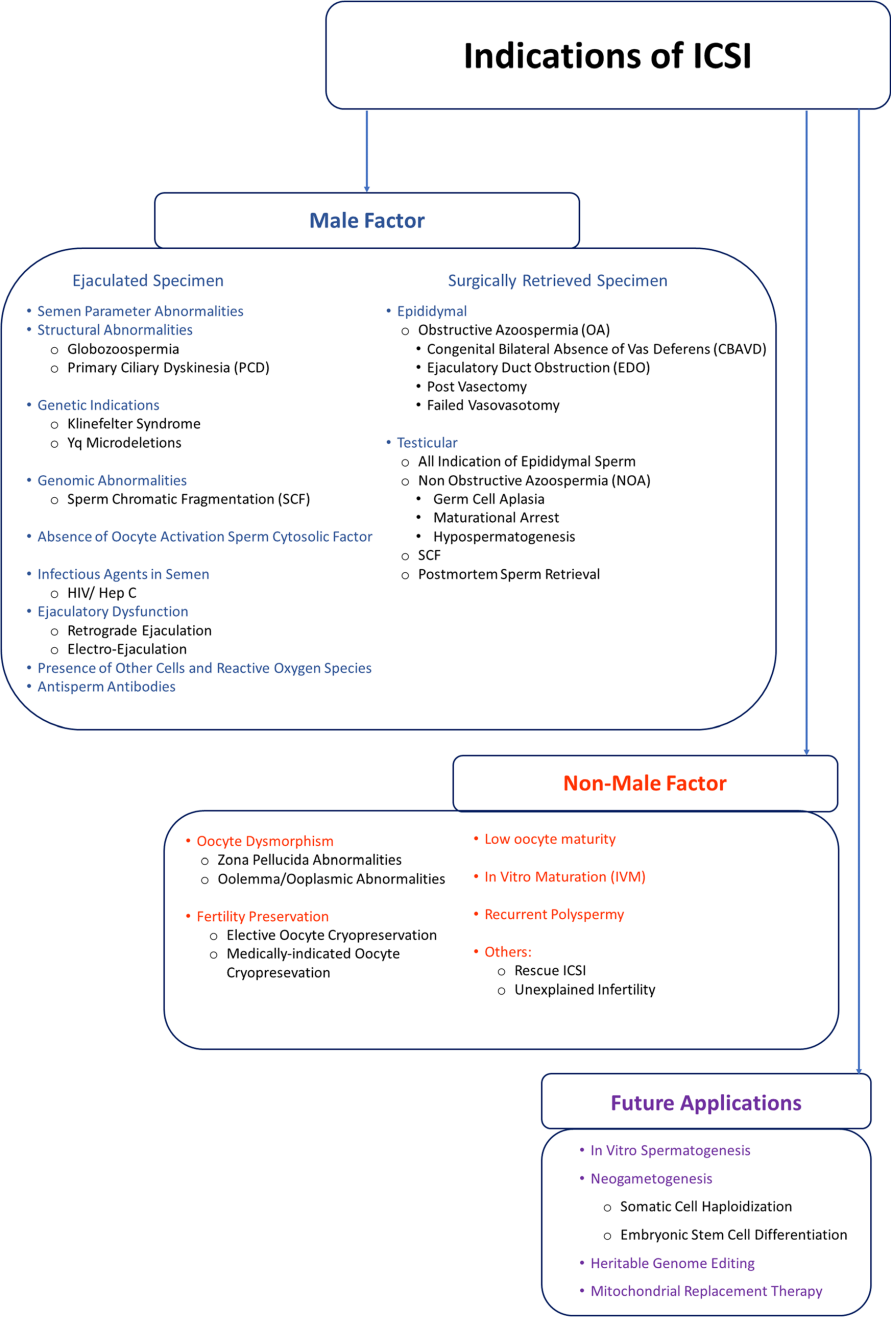
Indications of ICSI

How did ICSI manage to be the most widely used ART in the twenty-first century? In this manuscript, we describe the early micromanipulation techniques that led to the development of ICSI and its eventual widespread. In addition to describing its rise in popularity and expanding indications, we will discuss the potential future applications we envision for this versatile insemination method (Fig. 1).

## Early micromanipulation

The evidence that in vitro fertilization (IVF) was a viable option to infertile couples emerged in 1978, when the first live birth was achieved in a woman with bilateral tubal occlusion [4, 5]. This was the first birth by natural cycle IVF, i.e. it was achieved without the use of ovarian superovulation [6]. The use of unstimulated cycles, which yielded a 6% pregnancy rate, was used by all clinicians until 1982, when a study on human menopausal gonadotropin (hMG) was linked to an increased number of oocytes, reaching an average of 2.1–2.6 oocytes per retrieval and a clinical pregnancy rate of 30% [7].

Different attempts were made to improve pregnancy rates such as the transfer of the male gametes directly into the fallopian tubes, known as gamete intrafallopian transfer (GIFT). With this procedure, the oocytes retrieved laparoscopically were transferred in the fallopian tube together with spermatozoa [8]. This technique soon became obsolete because it was not indicated for women with tubal occlusion or male patients with compromised semen parameters. A variant of this technique was later introduced, zygote intrafallopian transfer (ZIFT), where the oocyte was fertilized in vitro and subsequently transferred to the fallopian tube, which was able to overcome these prior limitations. But very quickly, this method was found to be invasive, ineffective, and costly because of the need of 2 laparoscopic procedures; one for oocyte retrieval and one for zygote transfer [9]. This was particularly evident once the less invasive transvaginal aspiration of oocytes became the standard. However, among these patients, sudden unexpected fertilization failure started to emerge. This was later attributed to compromised semen parameters.

The routine utilization of conventional IVF was able to treat couples with tubal infertility, and soon was adapted to treat couples whose male partners had abnormal kinetic or morphologic characteristics of the spermatozoa, preventing the spermatozoa from penetrating the glycoprotein layer surrounding the oocyte with often failure to fertilize [10, 11]. The adoption of microdroplet insemination was proposed [12], entailing small drops of medium covered by oil loaded with pooled oocytes and high sperm concentration, but the results in fertilization rate were still poor and unpredictable. Different efforts to improve fertilization outcomes were made and the first attempts were focused on the manipulation of the zona pellucida (ZP), since it was initially hypothesized that the thick layer of glycoproteins would impede the penetration of the spermatozoon into the oocyte [13].

Therefore, an early experiment was done to completely remove the ZP in an attempt to facilitate the fusion of the male gamete with the oolemma but this resulted in high incidence of polyspermy. This approach was compromised by impaired embryo developmental dynamics [14] due to the loss of the ZP as a scaffold for the developing conceptuses. To overcome this obstacle, subsequent techniques aimed at thinning the ZP by the use of trypsin or pronase were investigated [15]. Despite the presence of sperm penetration in patients with previous total failure of fertilization, the aggressive enzymatic action resulted in embryos unable to cleave [15, 16]. Gordon and Talansky reported a procedure called zona drilling (ZD) in which an actual hole in the ZP was created by the action of acidified Tyrode’s medium delivered through a thin glass pipette to allow spermatozoa to bypass the ZP and interact directly with the oolemma. Although the fertilization rate improved to 32%, a high rate of oocytes damage was observed due to the acidic medium employed. Additionally, there was also an increase in the polyspermy rate since more spermatozoa were able to simultaneously enter the perivitelline space through the breach in the zona [13, 17].

Around the same time, Cohen et al. used mechanical means to produce a virtual opening in the ZP [10]. This approach was called partial zona dissection (PZD). Once the ZP was breached by a microneedle controlled by a micromanipulator, the oocytes were then conventionally inseminated. With this approach, the fertilization rate improved to 45% in some cases, but fertilization rates were still sporadic for patients with male factors due to severe asthenozoospermia. Also with this technique, polyspermy as high as 48% plagued clinical outcome [18].

In order to achieve fertilization while minimizing the polyspermy side effect, a later procedure known as subzonal insemination (SUZI) was advanced, where few spermatozoa were inserted in the perivitelline space with a micropipette [19]. This method was beneficial even when sperm concentration and motility were both compromised yielding more consistent results in comparison to ZD and PZD. Indeed, well-developed embryos were obtained, even in couples with a history of poor fertilization results. This was demonstrated in a study that involved 114 patients in which SUZI and IVF were performed simultaneously on sibling oocytes. The fertilization rate with SUZI was 39% versus the 6% obtained with IVF [20]. In another study on 43 couples (44 cycles) with fertilization failure after standard in vitro insemination, the fertilization rate and the cleavage improved after subzonal insemination, reaching 30.9% and 80%, respectively [21].

The early gamete manipulation techniques were proven successful in couples with mild male factor infertility, when only the parameters of the spermatozoa were impaired but purposeless in cases of gamete dysfunction [13]. Indeed, it was very important to select spermatozoa with normal morphology and intact acrosomes [22, 23].

**Fig. 2:**
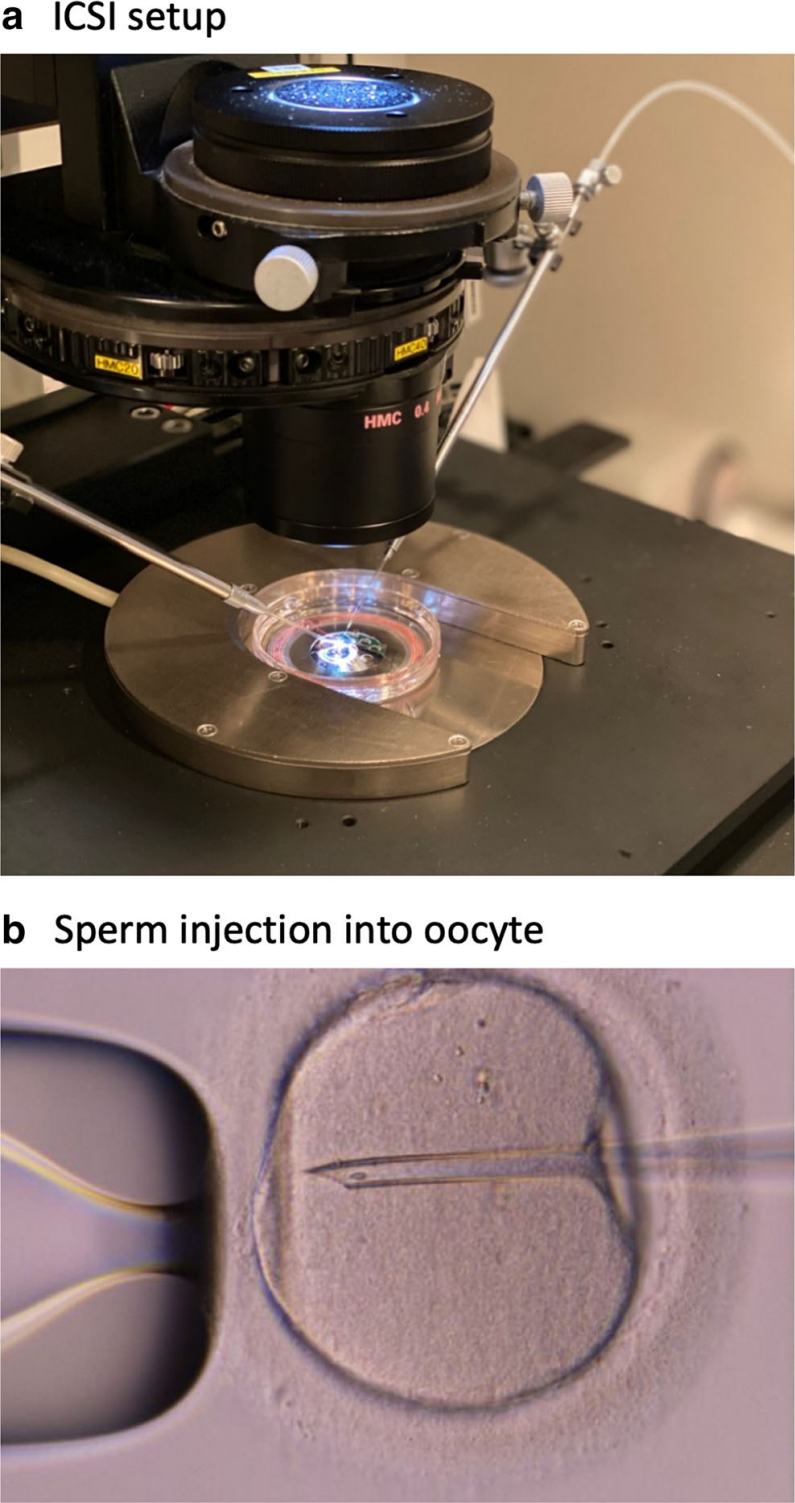
Intracytoplasmic sperm injection (ICSI) procedure. (**a**) The injection (right) and holding (left) pipettes are placed into micromanipulation tool holders on an inverted microscope and positioned using hydraulic joysticks. The ICSI dish is placed on the microscope stage. (**b**) The injecting pipette enters from the 3 o’clock portion of the oocyte and deposits the spermatozoon at the 9 o’clock portion of the ooplasm

In the late 1980s, different attempts were carried out by injecting the spermatozoon directly into the oocyte. The sea urchin [24, 25] and the Chinese hamster [26, 27] were the first species used to attempt these new procedures. Early on, a pregnancy was obtained in the rabbit [28] followed by a birth in the bovine species [29]. In 1987, this technique was applied in human oocytes as a proof-of-concept [30]. In a small series aimed at achieving pregnancy, it was shown that most oocytes injected were damaged, and the few that were fertilized were somewhat hindered in their ability to develop, resulting in failed implantation [31]. In spite of these initial disappointing results, and with a further refinement of tools and equipment, this procedure evolved in a new technique, intracytoplasmic sperm injection (ICSI; Fig. 2) [23]. This novel approach proved itself as the best way to treat male factor couples [32–34].

## Male factors indications of ICSI

### Ejaculated specimen

#### Semen parameter abnormalities

ICSI is the gold standard technique to treat male factor infertility. It was developed to enable the male gamete to bypass natural barriers surrounding the oocyte, such as cumulus cells, the zona pellucida, and the oolemma. It is because of its direct approach that ICSI is the most effective method to treat couples presenting with oligo-, astheno-, and teratozoospermia or a combination of all the above [33].

In cases of oligozoospermia, adequate fertilization rate, implantation rate, and clinical outcomes were observed when selecting apparently normal spermatozoa for ICSI [35]. In cryptozoospermia, previously considered azoospermia, spermatozoa cannot be observed in an ejaculated semen sample unless an initial centrifugation is carried out, followed by an extensive semen analysis. In those circumstances, ICSI is the only infertility treatment suggested for these men [36].

Cases of complete asthenozoospermia, where only immotile spermatozoa are retrieved, have an estimated frequency of 1 in 5000 men [37]. Completely immotile spermatozoa can be either viable or non-viable, and this distinction is not straightforward, requiring the addition of pentoxifylline, which has been linked to higher fertilization rates, allowing the identification of sperm cells with kinetic activity [38]. Other improvements of the sperm sample such as the use of theophylline and the sperm tail flexibility test (STFT) have been proposed, but are less effective than pentoxifylline [39–42].

Evaluation of sperm membrane integrity using the hypo-osmotic swelling test (HOST) has also been proposed. This test consists of adding spermatozoa to a hypo-osmotic solution and assessing the presence of swelling, which indicates an intact membrane and confirms spermatozoa viability [43]. HOST has been proposed to differentiate viable from non-viable spermatozoa in cases of necrozoospermia, a catabolic and degenerative condition that results in cell death and which is very often associated with asthenozoospermia. This allows a better selection of the spermatozoon to be injected with ICSI [44, 45].

On the other hand, the assessment of spermatozoa morphology is more complex and prone to inter-observer grading variability. Sperm morphology according to Kruger’s criteria has little to no prognostic value in ICSI outcome [46]. On the other hand, the identification under light microscopy of spermatozoa with normal shape of the head and detectable acrosomal structure, has allowed for good quality blastocyst development, even in samples with no normal form [46].

#### Structural abnormalities

##### Globozoospermia

Globozoospermia is a subtype of teratozoospermia characterized by round-headed spermatozoa with absent acrosomes, abnormal nuclear membranes, and midpiece defects [47]. In patients with partial globozoospermia, where some normally shaped heads are present, the selection of normal sperm cells for ICSI injection is appropriate. In complete cases, however, the utilization of assisted oocyte activation (AOA) can also achieve fertilization as in the case of 34 globozoospermic patients [48]. This disorder has been linked to genetic defects related to cytoskeleton organization, endoplasmic and Golgi network and acrosome formation [49]. In particular, recessive deletions and point mutations of three genes, dpy-19-like 2 (DPY19L2), spermatogenesis associated 16 (SPATA16) and protein interacting with C Kinase (PICK 1) result in globozoospermia phenotype. DPY19L2 is involved in sperm head elongation and development of the acrosome, SPATA16 is responsible for acrosome biogenesis, and PICK 1 plays a role in vesicular transport mechanism and acrosome biogenesis [50]. In addition, a homozygous mutation of aurora kinase C (AURKC), a gene involved in chromosomal segregation and cytokinesis, has been identified in large-headed spermatozoa [51]. ICSI, often supported by assisted gamete treatment or not, is the only viable option to obtain fertilization in globozoospermic patients [52].

##### Primary ciliary dyskinesia

Primary ciliary dyskinesia (PCD) is an autosomal recessive condition that is caused by a defect in the ultrastructure of motile cilia or flagella. The axoneme is the internal cytoskeleton of the flagellum or cilium, and is composed of a central pair of microtubule doublets, surrounded by 9 outer microtubule doublets, carrying the dynein arms. The sliding force of motility is provided by outer and inner dynein arms. A mutation can occur in any one of these structures, leading to abnormal beating of the flagella and often absent sperm motility [53, 54].

Kartagener syndrome is an autosomal recessive disease characterized by situs inversus and accounts for around 50% of PCD cases. Kartagener syndrome has been attributed to different gene defects affecting the dynein arms or microtubules structures, leading to structural abnormalities of *cilia* and *flagella*, disrupting their mechanism of action. A case report on a Kartagener syndrome patient where pentoxifylline was added resulted in the birth of a healthy infant after ICSI [55]. Other dynein arm defects have been reported, being associated with gene mutations affecting the axonemal dynein intermediate chain DNAI1, and the dynein heavy chain gene DNAH5 as well as other similar genes [56].

Collectively, the different types of sperm flagella abnormalities were previously known as dysplasia of the fibrous sheath (DFS). One of the first case reports of a live birth was in 1996; it describes a young man with a combination of DFS and dynein deficiency where ICSI performed with immotile spermatozoa led to a viable pregnancy and delivery [57]. Later on, a number of reports confirmed that ICSI was the only treatment option for most PCD patients, with its ability to overcome impaired motility and bypass the processes of natural fertilization [58–62].

In all of these forms of dyskinesia of the sperm flagellum, incubating sperm with pentoxifylline can seldom lead to motile spermatozoa [63] and should be attempted prior to injecting immotile spermatozoa.

#### Genetic indications

##### Klinefelter syndrome

Infertile men, particularly azoospermic men, are prone to higher chromosomal abnormalities, with an incidence of 12.6% [64], and with Klinefelter syndrome (47, XXY) being the most prevalent. Approximately 15% to 20% of Klinefelter syndrome (KS) patients are mosaics and present with two cell lines: 46XY/47XXY. Men with mosaic KS are reported to be more androgenized than non-mosaic KS, with lower estradiol and LH level in non-mosaic men [65].

KS is characterized by hypergonadotropic hypogonadism and intrinsic testicular failure, which in non-mosaic form, results in non-obstructive azoospermia (NOA). These men have spermatogonia at birth, but testicular function progressively deteriorates with onset of puberty, and is associated with a depletion of testicular germ cells [66]. However, even in the adult men’s testes, small, patchy distribution of spermatogenesis may still be present, and spermatozoa can be retrieved by testicular sperm extraction (TESE or microTESE). From the literature, we can argue that less than 10% of KS patients have sperm in their ejaculate while more than 90% will have to undergo TESE [67, 68]. When combined with ICSI, TESE can lead to a rate of pregnancy close to 50% (218 biochemical pregnancies over 410 ICSI cycles) and a live birth rate of 43% in KS [69].

As the first microTESE on KS patient was performed in 1996 [70], and the first pregnancy using TESE/ICSI in KS was reported in 1997 [71], until today there are still debates on whether to cryopreserve germ cells prior to reaching puberty and no clear clinical practice guidelines have been established yet [72]. Indeed, in a recent survey, 78% of practitioners agreed that testicular biopsy ought to be offered if no sperm is found in ejaculate, and more than 70% of endocrinologists, pediatric endocrinologists, and urologists promoted sperm freezing for KS patient between age 14–16 years [73].

##### Yq microdeletions

Y chromosome microdeletions are a condition associated with severe male factors infertility, and is seen in up to 18% of men with non-obstructive azoo- or oligozoospermia [74]. Different loci on the Y chromosome have been identified, and most of these genes have been located in a specific region known as the azoospermic factor (AZF) region, and found to have regional alterations (AZFa, AZFb, and AZFc). The most frequent microdeletion has been found to be AZFc (53–56%), followed by AZFa (4.7%) and AZFb (4.1%) [75, 76]. Furthermore, the chance of sperm retrieval for these patients based upon the specific region of Yq deleted differs. As a matter of fact, microdeletions of AZFa or AZFb regions are associated with no sperm retrieval except in rarer situations by TESE, while most men with AZFc microdeletions have sperm in the ejaculate, or can be retrieved from the epididymis and/or testis, and eventually be used with ICSI [77].

Furthermore, in the AZFa region, the USP9Y gene, an ubiquitin-specific peptidase 9 Y-linked gene, and the DDX3Y gene, which functions in RNA metabolism and translational regulation, have been identified. The partial or complete deletion of USP9Y causes severe oligozoospermia but spermatogenesis still occurs [78–80]. On the other hand, DDX3Y gene mutations or deletion is related to complete azoospermia, and spermatogenesis does not occur [79].

Through ART, and particularly with TESE/ICSI, men with Yq microdeletions and severe oligozoospermia or azoospermia have been able to reproduce. One study concluded that fertilization rates and clinical pregnancy rates were not significantly different from men with NOA with no Y chromosome microdeletions [81]. Moreover, evaluation of children of men with Yq microdeletions, born through ICSI, demonstrated that the inheritance of this defect has no effect on their psychological and physical development, except for a similar deletion in baby boys [82] and sometimes a different spermatogenetic profile in fathers and sons may ensue [83].

#### Genomic abnormalities

##### Sperm chromatin fragmentation

It has been recently recognized that an elevated sperm chromatin fragmentation (SCF) affects couples seeking procreation and can be one of the reasons to cause recurrent implantation failure and/or pregnancy loss [84, 85]. As mentioned, ICSI aims to select spermatozoa with adequate morphokinetic profile (normal morphology and motility) in order to inseminate oocytes, which may indicate that they are the spermatozoa with highest genetic integrity [86]. Directly compared to conventional in vitro insemination, ICSI yields a superior pregnancy rate in couples known to have high SCF [87]. Indeed, an algorithm of reproductive treatment is devised to use ICSI upfront to treat patients known to have high SCF, in an attempt to minimize futile ART cycles [88]. However, when clinical outcomes were compared between couples with high SCF and normal SCF, even ICSI outcome was affected by the extent of DNA damage in the ejaculated male gamete [89]. Specifically, ICSI results are negatively impacted by the high incidence of double-stranded DNA breakage resulting in delayed embryo development and poor embryo implantation due to high rate of embryo aneuploidy [90], while ICSI seems less affected by single-stranded DNA breakage [90]. In order to ameliorate the detrimental effect of such DNA insult, treatment can be attempted with the administration of Coenzyme Q10 which improves sperm parameters and apparently has an effect on the proportion of spermatozoa with an intact genome [91].

A more sophisticated alternative sperm selection method was proposed, aiming to select ejaculated spermatozoa with the highest genomic integrity and competence, such as testicular sperm extraction. By using microfluidic techniques together with ICSI, a higher rate of euploid conceptuses was achieved even in patients who had not been able to obtain suitable embryos to transfer in their historical cycles [92].

#### Absence of oocyte activation sperm cytosolic factor

ICSI has been able to greatly alleviate male factor infertility, but some rare cases of post-ICSI fertilization failure still occur at about 1–3%. In about 40–70% of unfertilized oocytes post-ICSI, the culprit has been attributed to a specific oocyte activating factor deficiency in the spermatozoa [93]. Evidence suggests that oocyte activation is triggered by a sperm factor, phospholipase C zeta (PLCζ) that, once in the ooplasm, activates a cascade of reactions leading to the release of Ca^2+^ in an oscillatory mode [94].

For these problematic cases of failed oocyte activation, assisted oocyte activation (AOA) can be carried out by exposing the oocytes, post-ICSI, to a chemical agent such as calcium ionophore or strontium chloride, or an electrical pulse [95, 96]. Treatment with one of these agents increases the Ca^2+^ permeability at the cell membrane, allowing an influx of extracellular Ca^2+^ into the ooplasma and thereby inducing Ca^2+^ release from intracellular calcium stores.

One pioneering study on AOA reported 17 couples with previously failed fertilization following ICSI. For these patients’ subsequent ICSI cycles, AOA was carried out by injecting the spermatozoon with medium containing a high concentration of CaCl_2_, followed by exposing the oocyte to calcium ionophore. As a result, couples with this peculiar defect were able to achieve an overall fertilization rate of over 70% [97].

Despite positive results, it is important to identify a sperm-related oocyte activation deficiency (OAD), or a sperm-related fertilization deficiency, before carrying out AOA to avoid unnecessary interventions. The demonstration of OAD is usually confirmed in couples with a history of fertilization failure with ICSI and a positive mouse oocyte activation test (MOAT), which measures the oocyte activating capacity of spermatozoa. If a MOAT test indicates a deficiency of the sperm oocyte-activating ability, then theoretically good results with AOA can be expected [98]. In a study carried out on 114 couples with history of poor or complete ICSI fertilization failure, male partners were initially screened using direct PLCζ assessment by immunofluorescence to determine whether poor fertilization was attributed to an oocyte- or sperm-related OAD [52]. If the PLCζ assay was negative, indicating an oocyte-related cause for fertilization failure, couples were treated in a subsequent ICSI cycle with a modulated stimulation protocol. Couples with a positive PLCζ assay, however, were further assessed with the MOAT to confirm sperm-related OAD. A deletion on the PLCZ1 gene in some of these men was also detected by DNA sequencing, confirming the absence of PLCζ. Deletions on PICK1, SPATA16, and DPY19L indicated an absence of the spermatozoa subacrosomal perinuclear theca, as observed in globozoospermic men. These couples underwent subsequent ICSI cycles with assisted gamete treatment (AGT), during which the spermatozoa and oocytes were briefly exposed to calcium ionophore. In both cases of oocyte- and sperm-related OAD, couples’ subsequent ICSI cycles yielded significantly higher fertilization and clinical pregnancy rates [52].

#### Infectious agents in semen

##### HIV/Hep C discordant couples

Assisted reproductive techniques may also benefit serodiscordant couples in which the male partner is infected by blood borne viruses such as HIV or Hepatitis C by preventing horizontal transmission. Conversely for seropositive females, intrauterine insemination (IUI) can be considered if other indications of female factor infertility are absent, and ICSI would be offered to avoid unexpected fertilization failure following potential IUI failure [99].

In seropositive males however, prerequisite highly active antiretroviral therapy (HAART), extensive sperm decontamination and ICSI are utilized in order to minimize horizontal and vertical virus transmission [100]. In fact, spermatozoa lack an HIV receptor and therefore the male gametes themselves are not infected as the virus is only present in seminal plasma [101]. Even though sperm processing by density gradient can yield viral-free samples safe for insemination and a seronegative offspring has been born from IUI using decontaminated spermatozoa from seropositive male [102], patients treated with HAART may present with impaired semen parameters including volume, concentration, progressive motility and compromised morphology [103]. In addition to an already impaired semen sample, proper sperm processing is required to remove excess viral particles, resulting in loss of additional sperm cells, leaving ICSI as the preferable insemination option to ensure successful treatment outcome [99, 104, 105]. To date, all infants born from ICSI with seropositive male parents tested seronegative [106].

#### Ejaculatory dysfunction

##### Retrograde ejaculation

Ejaculation is a complex process involving the sympathetic nerve fibers. The latter triggers the 2 phases of ejaculation: emission and expulsion. Failure of closure of the bladder neck during the expulsion phase results in reflux of semen into the bladder and is known as retrograde ejaculation (RE). Moreover, the spermatozoa recovered following urination are usually compromised by a non-physiological environment and appear non-motile. Performing IUI or conventional insemination would be unadvisable in such cases due to the impaired sperm kinetics and the possible exposure to toxic contaminants [107], but is still possible in specific cases with good concentration and motility and when adequate alkalization of urine is done by oral or intravenous administration of sodium bicarbonate [108].

In an early study by Nikolettos et al., the outcome of ICSI treatment in 16 couples with men presenting with RE reported 51.2% fertilization rate [109]. ICSI was able to generate live births using fresh spermatozoa retrieved from post-ejaculatory urine despite extremely low motility [110, 111]. Moreover, sperm retrieval directly from the bladder, known as the Hotchkiss procedure has been attempted. The use of cryopreserved spermatozoa retrieved from the bladder has yielded 62.6% ICSI fertilization, 37.5% clinical pregnancy rate and 28% live birth rate [112].

##### Electroejaculation

Men with neurologic impairment in their sympathetic outflow, as in demyelinating neuropathies (multiple sclerosis), traumatic spinal cord injury (SCI), or diabetes, often present with abnormalities or absence of ejaculation. Another common cause is psychogenic anejaculation (PAE). Medically assisted ejaculation methods for the treatment of these conditions are being used, such as penile vibratory stimulation (PVS), and electroejaculation (EEJ) by inserting a probe transrectally and sending an electric signal to stimulate ejaculation [113].

In an early study by Hovav et al., sperm retrieved by EEJ was assessed and shown to have decreased motility and normal or decreased concentration, thus demonstrating that EEJ can work and is a less invasive treatment option than surgical sperm retrieval [114]. Sperm retrieval by PVS or EEJ combined with intravaginal insemination or intrauterine insemination (IUI) should be offered to couples with abnormal ejaculatory processes as the first line of treatment. In the case of suboptimal motility or previously failed IUI with EEJ, ICSI should be used to maximize chances of fertilization and a successful pregnancy [115].

Moreover, studies on different methods of fertilization reported similar or even higher rates of pregnancy and delivery in EEJ/ICSI cycles when compared to EEJ/IUI cycles [116, 117]. To note that it is also possible to cryopreserve the spermatozoa retrieved from EEJ, with results as good as those with freshly obtained spermatozoa. With the use of frozen-thawed samples, the frequency of transrectal EEJ procedures can be thus reduced [118].

#### Presence of other cells and reactive oxygen species

Semen samples can contain cells other than spermatozoa, such as round cells (RC), and white blood cells (WBC),which can be accountable for reactive oxygen species (ROS) production. Presence of round cells in the ejaculate has been erroneously considered leukocytospermia. The origin of seminal RCs is not necessarily correlated to bacteriological growth. In this scenario, RCs are mostly immature germ cells with sloughed Sertoli cell remnants and indicate a damage on germinal epithelium usually caused by ailment and disrupted physiology.

Presence of seminal RCs correlates to a compromised sperm concentration, but more importantly, the sperm DNA fragmentation is also elevated when RCs are seen [119]. This results in an apparent impairment of fertilization and pregnancy rates with IVF but not with ICSI, even though a decreased blastocyst development has been observed in the ICSI group [120].

A male genital tract infection can occur in up to 15% of male infertility cases, and etiology has been attributed to genital tract infection affecting the urethra, epididymis and/or testis [121]. In this scenario, the presence of WBCs has been identified, and usually indicates the potential presence of bacteria in semen. However, leukocytospermia does not necessarily imply immunologic infertility caused by antisperm antibodies [122]. Indeed, the presence of white blood cells in male genital tract can cause elevation of (ROS), directly or indirectly, and may result in poor sperm motility and increase sperm DNA damage [123]. Therefore, leukocytospermia may cause male infertility by attenuating sperm kinetics and impairing male genomic integrity. Common treatment of leukocytospermia includes antibiotics therapy and antioxidant therapy [124]. Patients who underwent antibiotic treatment have shown recovered semen parameters, resolution of leukocytospermia, and increased pregnancy rate [125]. However, seminal leukocytes may not be used as a predictor for ART treatment outcome, as patients with or without WBCs in their semen were comparable, whether treated by standard IVF or ICSI [126, 127].

The dogma on leukocytospermia has been challenged in a recent paper using ploidy markers, protamine assays, and Sertoli cell cytoplasm markers. It was determined that leukocytes made up at most 26.7% of the round cells in the samples of all men assessed [119]. The authors were able to conclude that the etiology of round cells is actually the presence of nuclei of immature germ cells encased in Sertoli cell cytoplasm sloughed from the germinal epithelium due to an insult in the genital tract. These samples also exclusively had negative bacterial cultures. This in part explains why samples presenting with seminal RCs are associated with a compromised sperm concentration and normal morphology. ICSI is the ideal treatment method in these cases not only because it can take care of a semen sample with acutely compromised semen parameters, but also with an increase in SCF due to exposure to ROS generated from these ancillary cells. The selection of spermatozoa for ICSI through a viscous medium also prevents eventual bacterial and viral contamination, even if initially present in the ejaculated specimen.

#### Antisperm antibody

Antisperm antibodies (ASA) are immunoglobins directed against sperm antigen, which are positive in 5–15% of infertile men and 1–2% of fertile men [128–131]. ASA is usually IgG isotype when found in blood or lymph node, and in the form of IgA when in seminal secretions [132]. To date, the IgG-mixed anti-globulin reaction (IgG-MAR) test is the most widely used test for ASA detection, with positive results ranging from 2.6% to 12.9% depending on the threshold chosen for positivity [133].

The presence of ASA in either male or female partners is considered a type of immunological infertility. Since human semen is made up of spermatozoa, non-sperm cells, seminal vesicles and small molecules, the female genital tract may be sensitized and thus trigger local or systemic immunological responses. The immunological rejection of semen in the female genital tract is also known as failure of natural tolerance, and may lead to sperm elimination [134]. Within the female reproductive tract, ASA may hinder pre/post-fertilization processes at different levels including sperm agglutination, reduced sperm motility, impaired capacitation and acrosome reaction, inability to bind and penetrate ZP, failed sperm-oocyte fusion, and eventual unsuccessful embryo implantation. In those cases, standard IVF which relies on natural binding between the spermatozoon and oocyte is not an ideal treatment method and has been shown to yield reduced fertilization, pregnancy and live birth rates [135]. Most of the ASA is found to be on the head of the spermatozoa, thus preventing the acrosomal reaction and ability of the sperm head to penetrate into the oocyte [136, 137]. By directly injecting the sperm in the ooplasm, ICSI bypasses this obstacle.

In men with positive seminal ASA, semen parameters may be negatively affected including a reduction in total sperm count, concentration, motility, and viability [138]. However, sperm genomic integrity is not affected by ASA and therefore genomically healthy spermatozoa are still achievable [139], although proper sperm process using chymotrypsin-galactose may improve treatment outcome of conventional IVF by shedding antibodies bound to the spermatozoa [140]. ICSI, however, can easily bypass pre-fertilization biological hindrances caused by ASA. In fact, ICSI has shown to be an effective technique that yields comparable clinical outcomes as those cases with negative ASA, and the presence of ASA does not show any negative impact to ICSI outcomes [141, 142].

### Surgically retrieved spermatozoa

#### Epididymal

As aforementioned, ICSI requires only a single spermatozoon to fertilize an oocyte in spite of impaired kinetics, dysmorphism and maturational stage of the male gametes. Therefore, ICSI is regarded as the only method of insemination for men with azoospermia that require surgical intervention to retrieve spermatozoa. While azoospermia is observed in 1% of all men and 10–15% in males seeking fertility treatment [143], epididymal sperm retrieval is an efficient method to obtain a sufficient number of male gametes in men with obstructive azoospermia (OA) and a normal hormonal profile [144].

Obstruction of the male genital tract can present at different levels. Ejaculatory duct obstruction (EDO) occurs in the distal level and can be corrected by transurethral resection of the ejaculatory duct (TURED). In 50–70% of cases, spermatozoa are found in the ejaculate after the TURED procedure, but in 20% of cases, complications such as urine reflux, hematuria, urinary tract infection, or hematuria can occur [145]. Indeed, sperm retrieval techniques such as percutaneous and microsurgical epididymal sperm aspiration (PESA and MESA, respectively) can be used as alternatives to resection. .

Epididymal sperm retrieval can also benefit patients who have obstruction at the deferens vasa level. The etiology of vasal obstruction can be either congenital or acquired. Congenital obstruction may occur unilaterally or bilaterally (CUAVD or CBAVD, respectively). The genetic cause of CBAVD is mostly linked to a cystic fibrosis transmembrane conductance regulator (CFTR) mutation, and the symptoms account for 25% of the OA population [146]. CBAVD is present in 1% of infertile men and in up to 6% of men with OA [147]. Surgical sperm retrieval for OA patients using ICSI has a success rate of 96–100% regardless of the etiology of OA [144]. However, epididymal sperm retrieval is only meaningful or effective in OA patients with conserved spermatogenesis. Acquired vasal obstruction can be attributed to vasectomy and failed vasectomy-reversal including vasovasostomy and vasoepididymostomy [148] as well as other iatrogenic causes [144].

While obstructive azoospermia (OA) on the ductal system is accountable for 40% of azoospermia cases [149], the most common cause of OA is vasectomy as a method of elective sterilization. Approximately 500,000 vasectomies are performed in the United States annually [150, 151]. ICSI is the sole and trusted method to utilize epididymal spermatozoa in ART.

#### Testicular

Testicular sperm extraction (TESE) offers a direct path to obtain male gametes directly from testes, and it can be utilized for OA patients as well, as it covers all the indications of the use of epididymal sperm. In order to obtain male gametes for males afflicted by non-obstructive azoospermia (NOA), surgical sperm retrieval directly from the germinal epithelium is required to gather these precious gametes. NOA can be further divided into 3 categories: hypospermatogenesis, maturational arrest and germ cell aplasia. As previously stated, ICSI bypasses the natural barriers between male and female gametes, and indeed, testicular spermatozoa can only be utilized in conjunction with ICSI due to the low maturity of male gametes [152].

##### High sperm chromatin fragmentation

While surgical sperm extraction is mostly performed on azoospermic men, utilization of those relatively immature gametes may benefit couples who present with elevated sperm chromatin fragmentation (SCF). High SCF may result in poor fertilization and recurrent pregnancy loss and can occur in men with normal or even optimal semen parameters [153]. In a recent study., a progressively decreasing level of SCF was observed when spermatozoa are retrieved from proximal levels of the male genital tract. Even though testicular spermatozoa have the lowest level of SCF due to lower exposure of oxidative stress, epididymal spermatozoa outperform the more immature gametes from germinal epithelium in clinical cases as they offer a balance between gametal maturity and SCF [154]. Conclusively, surgical sperm retrieval in conjunction with ICSI is a preferred approach to efficiently treat couples plagued by high SCF.

##### Post mortem sperm retrieval

Although the application may be legally, ethically and morally challenging, postmortem sperm retrieval (PSR) by methods such as testicular and epididymal resection, testicular biopsy and vasal aspiration can be offered to obtain and preserve the still viable gametes from a man who has been pronounced brain dead [155]. Needless to say, viable gametes can be extremely rare when the PSR is not conducted immediately. Therefore, in order to grant fertilization using these extremely precious specimens, ICSI is the preferred method of insemination.

## Non-male factor indications of ICSI

### Oocyte dysmorphism

#### *Zona pellucida abnormalities*

Following standard in vitro insemination, it has been evidenced that abnormalities of the zona pellucida (ZP) may affect fertilization, embryo development and clinical outcomes [156]. Lack of a ZP protein, ZP dysmorphism (thinned, thickened, irregularly shaped ZP), and/or complete absence of the ZP are alterations that have been demonstrated [157].

All mammalian ZPs consist of 3 sulfated glycoproteins, ZP1, ZP2, and ZP3, with human oocytes containing an additional ZP4. In humans, the protein that acts as the primary sperm ligand and acrosome reaction-inducer during fertilization remains controversial [158, 159]. However, one generally accepted mechanism is that the spermatozoon binds to ZP3 protein, also known as the sperm-recognition protein, after which it undergoes the acrosome reaction and binds to ZP2 protein, to penetrate the ZP [157]. Oocyte maturation defects and consequent female infertility have been linked to variants in ZP1 and ZP3 proteins [160, 161]. Moreover, a study was conducted to assess abnormal ZP phenotype and total fertilization failure following standard in vitro insemination in 2 unrelated infertile women, with a family history of consanguinity. They were found to have no ZP2 proteins, resulting in only the presence of a thin zona layer,. Despite a thin ZP, the lack of ZP2 protein was linked to defective sperm binding and penetration, as well as fertilization failure. This demonstrates that a ZP2 protein pathogenic variant also causes in vitro fertilization failure, and it is only after direct injection of sperm into the ooplasm that fertilization and pregnancy occur [162].

Another common abnormality is an alteration in the structural integrity of the ZP, which results in a frail structure that breaks easily with manipulation. Studies have reported higher than expected rates of fertilization failure using standard in vitro insemination [163]. This has actively stimulated the development of different assisted fertilization techniques aimed at bypassing the ZP and resulting in the development of ICSI. Indeed, a clinical trial demonstrated improved fertilization and pregnancy rates following ICSI in 92 patients with fertilization failure in their first IVF cycle, 28 of which had abnormal ZP morphology [164]. Also, by using modified laser assisted ICSI to inject these oocytes, the zona was thinned, and sperm was injected through the thinned area. This approach, which combines less invasive ICSI with assisted hatching, improves oocyte survival [165]. Later, a study on 212 ICSI cycles, where patients were randomly divided between laser assisted ICSI and conventional ICSI, demonstrated higher fertilization and pregnancy rates with laser assisted ICSI [166].

While ICSI has resolved the issue of overcoming the zona layer, it requires the removal of cumulus cells, which has evidenced the emergence of other defects of these oocyte coats. Indeed, during the denudation procedure in preparation for ICSI, the ZP can be occasionally damaged, leading to zona-free oocytes. In this regard, a retrospective comparison was done between 135 zona-free oocytes (oocytes where Paws damaged and subsequently removed) and 216 zona-intact oocytes. The results showed that the zona-free oocytes were successfully fertilized following ICSI, and there was no significant difference in cleavage, blastocyst formation, or survival rate between the zona-free and zona-intact oocytes [167]. A study on zona-free embryo culture concluded that although beneficial, the presence of the zona does not seem to be indispensable to all stages of oocyte and embryo development in vivo [168]. Indeed, follicle development, oocyte maturation, fertilization, and embryo development in vivo still occur in zona-free mammalian oocytes. Under laboratory conditions, the zona seems to be a passive, artificially hardened layer that from fertilization to the blastocyst stage envelopes the embryo [168]. Studies with domestic animals demonstrated that zona-free zygotes can be cultured in isolated systems (as individual droplets), without compromising blastocyst formation rates and quality [169, 170]. Zona-free culture may become a viable option for zona-free oocytes in humans.

These clinical evidences confirm that ICSI can successfully fertilize oocytes with damaged or absent ZP, giving a chance to patients with low egg yield and structurally compromised oocytes to have a normal fertilization followed by normal blastocyst development with fair chances of pregnancy.

##### Oolemma/ooplasmic abnormalities

It is commonly accepted that a morphologically healthy oocyte is one that is at metaphase II (MII), with a moderately granular, vacuole-free cytoplasm, a thin perivitelline space, and a non-fragmented round polar body [156]. In addition to the zona pellucida, a second obstacle that spermatozoa are faced with is the oolemma. In order to penetrate the oolemma, the sperm has to be fully capacitated and contain an acrosome with functional post acrosomal segments. While the presence of an oolemma receptor has been disputed for a long time, recently, a protein identified as IZUMO has been described. IZUMO 1 is a ligand found on the spermatozoa membrane that binds with JUNO, a folate receptor anchored on the egg oolemma [171]. Following fertilization, JUNO is rapidly shed from the egg membrane, contributing to the block of polyspermia at the oolemma level [172]. In this instance, ICSI presents itself as the only technique to bypass this hurdle.

On the other hand, oocyte dysmorphism can be characterized by an abnormal cytoplasm (granular, or dark), cytoplasmic inclusions (vacuoles, smooth endoplasmic reticulum, or refractile bodies), abnormal perivitelline space, abnormal polar body (giant, duplicated, fragmented) or non-spherical shapes [173]. Several studies have tried to investigate the ability of dysmorphic oocytes to fertilize. One pioneer study showed that when abnormal oocytes were conventionally inseminated no fertilization occurred, whereas the use of ICSI improved fertilization and clinical outcomes [174, 175]. These aberrations in the morphology of oocytes have little or no consequences on the fertilization and cleavage rate post-ICSI.

Abnormal morphological features are multifactorial; they can be due to the superovulation protocol, the nature of the gonadotropic drugs used (natural or synthetic), or the hormonal environment generated by these drugs. Different studies linked causative agents to each defect seen [173, 176, 177]. Furthermore, a meta-analysis on the effect of oocyte morphological characteristics in relation to clinical outcome concluded that, based on the data from eligible studies, MII oocytes with a large polar body, large perivitelline space, refractile bodies, or vacuoles were associated with an impaired fertilization rate with standard in vitro insemination. However, there was no significant effect on the embryo quality [178].

On the other hand, in the setting of ICSI, it is possible to evaluate the morphology of the oocyte immediately after removal of the cumulus-oocyte complex (COC), whereas standard in vitro insemination is limited at the evaluation of the COC. It is only the following day, after the inseminating spermatozoa have dispersed the cumulus cells from the surface of the oocytes, that a detailed morphology can be done, at which point the oocyte has already been fertilized or not. A study was conducted to assess the effect of centrally located cytoplasm granulation (CLCG) on ICSI outcome in terms of embryo cleavage, ongoing pregnancy, and live birth rate. These oocytes with CLCG were retrieved from women with a high pesticide exposure. The higher the density of the CLCG, the lower the pregnancy rate. This was presumably due to a dysregulated intra-ovarian microenvironment that, during oocyte maturation, affects oocyte morphology with consequent impairment of clinical outcome. The author opted to use ICSI to be able to “read” the oocytes’ cytoplasm and overcome the CLCG, granting fertilization [179]. Similarly, other studies confirm ICSI as the preferential tool to inseminate oocytes that require detailed evaluation of their morphological features [180–183].

#### Fertility preservation

##### Elective oocyte cryopreservation

Although both embryo and sperm cryopreservation has been well-established, it is only relatively recently that oocyte cryopreservation has emerged as an additional fertility preservation option for women. Oocyte cryopreservation in combination with ART provides several advantages for women anticipating age-related fertility decline, elective cryopreservation when a designated partner has not been identified, or cancer patients prior to gonadotoxic cancer treatment.

The first pregnancy from frozen-thawed oocytes was reported in 1986 [184] and since then, cryopreservation of oocytes has cemented its position in assisted reproduction. Standard in vitro insemination was useful in this anecdotal case achieving 83% fertilization, and a cleavage rate of 60%. With standard in vitro insemination as the obvious method of insemination at that time, all the frozen-thawed oocytes in the early 1990s were inseminated by this method. Successful fertilization and embryo development were obtained worldwide. However, unpredictability of egg survival and most importantly scant fertilization limited the use of this procedure. It was the report of the first Italian experience with slow freezing in combination with ICSI that produced the first live birth after intracytoplasmic sperm injection of a cryopreserved oocyte in 1997 [185]. With the utilization of ICSI, different studies and clinical trials were conducted to compare the treatment outcomes of standard in vitro insemination and ICSI on thawed-frozen oocytes [186] and early on, it was found that ICSI was able to rectify fertilization issues caused by a hardened ZP [186–188].

Due to its consistency in granting promising fertilization outcomes in the past two decades, ICSI has been the tool used to inseminate these oocytes and for experiments on cryopreservation,. Indeed, studies on oocyte cryopreservation were done in Italy, where there was a legally imposed limit of 3 inseminated oocytes per cycle. In one study, the author randomly divided the cycles into 2 groups: 120 autologous IVF cycles using vitrified oocytes and 251 cycles using freshly retrieved oocytes [189]. They concluded that in terms of fertilization and embryo development rates, there was no difference in ICSI performance between fresh or frozen oocytes. At the same time, a similar study also showed no difference in one group of freshly inseminated oocytes by ICSI compared to another group of cryopreserved oocytes [190]. With the emergence of supernumerary oocytes and to avoid discarding the remaining oocytes, oocyte banking has now become a routine practice and insemination of the survived oocytes post warming procedure is done by ICSI.

Moreover, in studies conducted to compare 2 methods of cryopreservation, slow-freezing versus vitrification, the ability of the cryopreserved oocytes to fertilize after thawing was evaluated only by ICSI [191–193]. Fertilization outcomes and pregnancy rates of previously cryopreserved oocytes were evaluated using ICSI as the method of insemination in many more studies [194–199].

Recently, an evidence-based counseling tool was created to guide physicians and women desiring elective oocyte cryopreservation; it estimates the number of oocytes to freeze and predicts the likelihood of having one, two or three live birth(s) based on the patient’s age and the results of ICSI cycles in their patient population [200].

##### Medically-indicated oocyte cryopreservation

Fertility preservation prior to gonadotoxic cancer therapy is becoming highly promoted in most oncology centers to preserve the reproductive potential in cancer survivors. Also, the survival rate for cancer patients has dramatically improved in the last two decades, pressing the need for cryostorage of these precious gametes. Oocyte cryopreservation has been preferred over embryo cryopreservation, particularly in single women refusing sperm donation and when embryo freezing is avoided for ethical or religious issues, or prohibited by law [201].

One of the first oocyte cryopreservation cycles for a cancer patient before her treatment was reported in 2007. By using ICSI the cycle yielded a successful live birth through a gestational carrier [202]. In a retrospective study on 357 women who had their oocytes cryopreserved after being diagnosed with cancer, 11 women returned for assisted reproduction. Their oocytes were fertilized by ICSI and 4 pregnancies and live births were reported [203]. Additionally, in a single center observational study, women with different types of cancer underwent oocyte retrieval cycles for cryopreservation prior to their treatment. After fertilization by ICSI, the pregnancy rate was 36.4% and delivery rate was 18.2%, demonstrating that oocyte cryopreservation combined with fertilization by ICSI is a good opportunity for fertilization preservation [204]. Finally, even if cancer patients were described as having a poorer response to ovarian stimulation [205], it was demonstrated that all the delivered babies were healthy and at term [206]. The hope of having a family after a cancer diagnosis can contribute to better acceptance of the oncologic treatment.

#### Low oocyte maturity

In earlier years, standard in vitro insemination was the only available method to treat couples with a history of low fertilization, or complete fertilization failure. These poor outcomes were largely attributed to the number of follicles, oocytes retrieved, and proportion of mature oocytes [207]. In an ART protocol, the aim is to harvest healthy mature oocytes in order to increase chances for a successful pregnancy. Although oocyte maturity can be assessed with difficulty and only by skillful individuals in the presence of the cumulus cells [208], oocyte nuclear maturity is only properly assessable after the removal of the cumulus cells and can be identified by the extrusion of the first polar body (PB) in the perivitelline space, indicating a successful completion of meiosis I and arrest at the metaphase II (MII) stage. Failure to yield an optimal maturation rate entails that some oocytes remain at either the germinal vesicle (GV) or metaphase I (MI) stages [37]. Thus, the proportion of MII oocytes retrieved can be affected by follicle size, ovarian stimulation protocols, or the surgeon’s ability to retrieve the smallest antral follicle [209]. An assessment of couples with low fertilization found that standard in vitro insemination was not beneficial in achieving higher fertilization rates in subsequent cycles [210].

The introduction of ICSI, however, was able to provide an effective alternative for couples with recurrent poor fertilization. A study on 38 couples with a history of low fertilization (<25%) compared IVF and ICSI outcomes on sibling oocytes and confirmed that while IVF treatment could not provide any improvement, ICSI was able to achieve a significantly higher fertilization rate [211]. The utilization of ICSI in these cases is helpful in order to assess the actual number of mature oocytes prior to injection, and therefore provide time for further in vitro maturation of the remainder GV and MI oocytes. Even with ICSI, however, the proportion of mature oocytes of the entire retrieved cohort influences successful outcome. Indeed, a recent study evaluating oocyte maturity allocated 7672 ICSI cycles according to their oocyte nuclear maturity ratios. The minimal maturation cohort, characterized by the highest proportion of MI and GV oocytes, displayed significantly lower normal 2PN fertilization, implantation, and clinical pregnancy rates, indicating an association of the low number of mature oocyte within the cohort with oocyte readiness to be fertilized [209]. The utilization of ICSI would allow for a more detailed information on the response of a patient to a specific superovulation protocol.

Although nuclear maturity can be morphologically validated by the extrusion of the first polar body, evaluating for the presence of a spindle has proven to be linked to more promising outcomes. In a recent study, polarized light microscopy was used to appraise oocyte maturity by looking at the presence or absence of a spindle, in addition to the polar body [212]. The authors considered that incomplete maturity is often seen in cases where there is a polar body, but no spindle. By removing the cumulus cells in the setting of ICSI, they could confirm complete maturity and allow them to adjust the timing of injection. Results showed that, of the examined oocytes retrieved from poor/slow responding patients with low oocyte yield, 32.6% did not display MII spindles. However, by adjusting the timing of ICSI insemination by lengthening the in vitro culture of the oocytes, successful fertilization, embryo development, and live births were observed [212]. Therefore, the ability of ICSI to enhance fertilization chances by direct spermatozoon injection makes it the most appropriate treatment in cases with low oocyte maturity. Moreover, the utilization of ICSI has allowed investigation into the contribution of ooplasm maturity on ART outcome. In a retrospective analysis, the duration of time interaction between oocyte and cumulus cells was lengthened, allowing the ooplasm to reach a proper maturity granting successful fertilization even with ICSI [213].

#### In vitro maturation

In vitro maturation (IVM) was originally developed as a variant to traditional in vitro fertilization (IVF) and was addressed to women with polycystic ovarian syndrome (PCOS), prone to ovarian hyperstimulation syndrome (OHSS), or other factors preventing them from undergoing a typical ovarian superovulation [214]. In IVM procedures, oocytes are retrieved from small and intermediate sized follicles when the largest of the cohort has not yet surpassed 13 mm in diameter. These cumulus-enclosed oocytes are then matured in vitro prior to insemination by small dosage gonadotropin, with or without hCG [215, 216].

Current literature on IVM in humans focuses mainly on its effectiveness or related culture conditions [217–219], with few studies comparing different insemination protocols. One such study included 8 PCOS patients treated in 8 IVM cycles where sibling oocytes were equally inseminated by IVF or ICSI. No significant differences in fertilization, blastocyst development, or clinical pregnancy rates between the two insemination methods were found [214]. Another assessment, however, found that 138 in vitro-matured oocytes fertilized by IVF yielded significantly lower fertilization than 151 oocytes fertilized by ICSI, although cleavage rates remained unaffected [220].

While the debate of using one insemination method versus another has not yet been settled due to the somewhat inconsistent fertilization of IVF in IVM cases, ICSI is widely considered to be the preferred procedure as it provides higher chances of yielding successful fertilization [221].

An assessment of 324 in vitro-matured GV oocytes and 341 MI oocytes found that, while IVF and ICSI yielded comparable normal fertilization rates in the GV and MI-derived oocytes, the occurrence of multiple pronuclei formation in the MI-derived oocytes was significantly more frequent when standard IVF was used [222]. These findings suggest that some in vitro*-*matured oocytes are more prone to allow polyspermy and therefore exhibit signs of abnormal zona behavior. Indeed, electron microscope assessment has evidenced that oocytes in culture media are at different stages of maturation, with specific morphokinetic characteristics [216]. Ultrastructural changes and altered properties of the ZP have been observed in in vitro*-*matured oocytes, and cumulus cells surrounding the abnormal oocytes have been shown to possess high apoptotic potential, which potentially leads to higher oocytes degeneration rates [216]. Moreover, a recent morphokinetic analysis of in vitro*-*matured oocytes using time-lapse microscopy found that early key developmental events, such as polar body extrusion and pronuclear fading, were significantly delayed following fertilization [223].

We also cannot exclude a potential negative effect of high hormone levels on in vitro-matured oocytes. In a retrospective analysis of 703 patients undergoing IVF, high serum FSH was found to be associated with brown ZP of oocytes, as well as impaired fertilization, embryo quality, and pregnancy outcomes [224]. Another study revealed that culturing oocytes in high doses of gonadotropins compromised their mitochondrial membrane potential, and resulted in the downregulation of genes involved in spindle formation and cell cycle control [225]. Regarding the use of protein supplements in IVM culture medium, an evaluation of bovine oocytes found that the addition of BSA-V, fetal calf serum, or polyvinyl alcohol during IVM hindered oocyte nuclear maturation and yielded a significantly lower number of morulae and blastocysts [226]. However, embryonic development rates were restored when BSA-V was replaced with purified BSA, indicating that the effect of FSH on oocyte nuclear maturation may be influenced by substrates present in the IVM medium.

As demonstrated by these studies, the success of IVM is dependent on the ability to overcome lags in nuclear and cytoplasmic maturation. Therefore, insemination at the precise time of maturation is crucial to provide optimal chances of fertilization. This can only be achieved by individual sperm penetration unconstrained by the zona characteristics, thus validating the use of ICSI for IVM.

#### Recurrent polyspermy

Standard in vitro insemination was designed to treat couples unable to achieve a pregnancy by natural means. However, this procedure, was often associated with multiple sperm penetration resulting in abnormal fertilization. Genetic analysis of embryos derived from multi-sperm fertilization has shown the spermatozoon to be the gamete responsible for carrying the centrosome. Functioning as a scaffold for the mitotic spindle and located near the nucleus, the centrosome in the zygote controls the first mitotic division. This underlines the importance of only a single spermatozoon with the oocyte for subsequent normal zygote development [227].

An early study found that 12.2% of fertilized oocytes obtained from 67 IVF cycles stimulated with human menopausal gonadotropin (hMG) were polyspermic but cleaved into normal appearing 2- and 3-cell embryos. They argued that corona-cumulus-removal at 15–18 h post standard in vitro insemination, instead of 40 h, is therefore essential determining the occurrence of polyspermy and avoiding the potential transfer of a diploid conceptus. [228]. Similarly, another study found 10% of oocytes to be polyspermic after standard in vitro insemination, but by adjusting the insemination time according to oocyte maturity and by decreasing the concentration of spermatozoa, the polyspermy rate decreased [229]. Higher rates of polyspermy have also been linked to high estradiol levels prior to hCG administration [230].

Most 3PN zygotes after standard in vitro insemination are of dispermic origin, with two centrosomes. The development of ICSI, however, led to the observation of a new pattern of abnormal fertilization: digynic 3PN zygotes consisting of 3PN and a single polar body (PB), with only centrosome. Due to partially-removed cumulus cells or a fragmented PB, digynic 3PN zygotes were overlooked in standard in vitro insemination [231].

Additionally, 3PN zygotes resulting from ICSI are largely due to failure of the oocyte to complete the second meiotic division after sperm injection, resulting in a third pronucleus from an unextruded PB [232]. Experiments comparing 3PN IVF zygotes (oocytes fertilized by standard in vitro insemination) and 3PN ICSI zygotes showed that karyotypes of 3PN ICSI embryos were closer to euploid than 3PN IVF embryos, indicating that the number of centrosomes in a 3PN zygote affects the integrity of the karyotype [233, 234]. The use of ICSI is able to eliminate dispermic triploidy even if it does not prevent oocyte-induced 3PN formation. .

Nevertheless, the incidence of digynic 3PN zygotes after ICSI is only 3.3% and their occurrence is related to superovulation protocol response of a particular patient to a specific gonadotropin regimen, higher estrogen level on day of hCG administration, or number of oocytes retrieved [233–235]. The appearance of 3PN embryos after ICSI is not, however, associated with lower pregnancy rates [236], although a high incidence rate of 3PN zygotes after ICSI is a negative predictor of fertilization and implantation rates for the remaining normally fertilized oocytes [235, 237].

Adjusting superovulation protocols or other risk factors may better control the risk of 3PN zygotes, but because ICSI outcome is highly reliant on oocyte quality, 3PN zygotes in ICSI cycles may signal an occult oocyte factor that could reflect oocyte competence [238], and require further investigation and appropriate treatment. ICSI is, thus, an ideal treatment for patient affected by recurrent polyspermy with standard in vitro insemination as it prevents multiple sperm fertilization and can help determine the cause of 3PN zygotes.

#### Other

##### Rescue ICSI

Early attempts of re-inseminating oocytes that had failed standard in vitro fertilization involved re-inseminating them in vitro or by partial zona pellucida dissection (PZD), and proved that in spite of enhanced fertilization, it generated an increase in polyspermia rate [239]. The concept of rescue ICSI surfaced in 1993, when in 23 patients, 115 MII one-day-old oocytes failed to fertilize with standard in vitro insemination were re-inseminated by ICSI. The fertilization and cleavage rate were 38% and 82% respectively following this attempted ICSI [240]. Another study compared oocytes that after standard in vitro insemination had a fertilization rate of 64.3% to those that failed and were re-inseminated by ICSI, reaching a comparable fertilization rate of 60.2% [241].

By directly injecting a single spermatozoon, the initial implementation of rescue ICSI was to avoid polyspermy and bypass a thick, as well as dysfunctional, ZP which lacked appropriate sperm glycoprotein receptors that may have led to the fertilization failure in the first place. Rescue ICSI proved superior and more reliable than prior endeavors with multiple sperm re-insemination and PZD [242]. Nonetheless, the success of fertilization by rescue ICSI is offset by a slim pregnancy rate. This might be due to a difference in the maturity of the oocytes at the time of rescue ICSI [243].

Furthermore, the low implantation rate after intrauterine replacement of embryos generated by rescue ICSI may be related to a higher rate of aneuploidy explained in an earlier study [244] as being linked to oocyte abnormalities due to a prolonged incubation.

A proof-of-concept to enhance rescue ICSI efficacy, by preventing oocyte aging, was carried out by inseminating oocytes determined to have failed standard in vitro insemination only 9 h after oocytes retrieval. While the fertilization rate was significantly higher with rescue ICSI (90.2%), the pregnancy rate was significantly compromised (30%) [245]. Interestingly, this experiment of early rescue ICSI insemination was repeated more recently by shortening the time of ICSI even more, at 6 h post retrieval. This was carried out only in oocytes that the authors described as having a putatively abnormal pellucida. Although fertilization rate appeared higher, pregnancy rate again was hindered. Both these studies have a major flaw due to the initial assessment of fertilization which is defined by the absence of extrusion of the second polar body. That, together with the absence of any genetic evaluation, may overlook multiple sperm penetration [163].

##### Unexplained infertility

The diagnosis of unexplained infertility in a couple unable to reproduce is made after excluding common causes of infertility including male factors, ovulatory disorders, tubal damage, uterine or peritoneal abnormalities [246]. Many algorithms for the management of unexplained infertility have been proposed, and they vary mainly based on duration of infertility and parental age. One common point is that the approach to unexplained infertility is from conservative to more invasive interventions [247]. Overall, the two most applied treatments in patients with unexplained infertility are IUI and IVF (either standard in vitro insemination or ICSI). While IUI represents a simple inexpensive approach, it does not allow an understanding of stealth reasons for the inability of the couple to reproduce. Therefore, when IUI fail, the utilization of ART seems reasonable to investigate issues related to fertilization and embryo development.

Studies assessing the efficacy of IVF, and mainly ICSI, in patients with unexplained infertility are scarce. One study selecting couples with unexplained infertility and that failed IUI and were subsequently treated by ART, both by IVF and ICSI, concluded that there was no difference in fertilization rate and embryo developmental quality between standard in vitro insemination and ICSI; however there was no total fertilization failure in ICSI-inseminated oocytes [248]. In that case, for patients with unexplained infertility and failed IUI, the results would be more favorable if ICSI is used over standard in vitro insemination, to avoid a total fertilization failure with the latter technique. The same conclusion was reached in a prospective randomized trial on 60 women with unexplained infertility undergoing either standard in vitro insemination or ICSI, with a rate of fertilization failure of 6.7% in standard in vitro insemination and no case of failed fertilization reported in ICSI [249]. Even if the authors do not recommend the routine use of ICSI for these patients, the ability of this insemination method to prevent fertilization failure and increase the number of embryos carried a potential psychological benefit for the couple [207]. On the other hand, a multicentered randomized controlled trial comparing standard in vitro insemination and ICSI in cases of non-male factor infertility reported no advantage for ICSI in terms of clinical outcomes, having obtained higher implantation and pregnancy rates with standard in vitro insemination when compared with ICSI [250].

Additional comparative studies on the outcomes between ICSI and standard in vitro insemination in patients with unexplained infertility have demonstrated higher fertilization rates with ICSI [251–254]. In one of the prospective cohort trials, rescue ICSI on oocytes that had failed to fertilize by standard in vitro insemination was also able to fertilize 10.9% of these oocytes [251]. The use of ICSI has also been recommended for couples with unexplained infertility where the female partner yielded a limited number of oocytes (less than 6 oocytes retrieved per cycle) [252].

Finally, a retrospective review of ICSI cycles, including 407 cases with unexplained infertility and 651 cases with male factor infertility, revealed that ICSI achieved comparable clinical pregnancy and live birth rates independent of the diagnosis [255]. ICSI is widely considered the preferred method to level consistent fertilization and prevent failure.

## Future applications

Because of the versatility of ICSI, its large popularity, and ability of the technique, it is feasible to consider that this insemination procedure will be applied in new, upcoming technology that will fulfill the goal of neogametogenesis and the alleviation of genomic defects. A list of the more realistic forthcoming techniques has been listed in Table 1.

**Table 1:** Future applications

Goal	Technique	Utilization
Neogametogenesis	In vitro gametogenesis	ICSI is used to inject round spermatids and spermatozoa created in vitro*.*
	Somatic cell haploidization	ICSI insemination is the preferred method of insemination for artificial oocytes due to better fertilization and post-fertilization development [256–258].
	Stem cell differentiation	ICSI provides a means to overcome gamete fusing issues arising from immature spermatid-like cells and round spermatids created through stem cell differentiation [259].
Heritable gene defect	Heritable genome editing	ICSI would be required to deliver mRNA [260] and for the coinjection of spermatozoa with CRISPR-Cas 9 [261] to edit target genes.
	Mitochondrial replacement therapy	Due to the requirement of heavy oocyte manipulation, precise timing of insemination, and ability to transfer pronuclei, spindles, and polar bodies, ICSI would be the only option for this experimental treatment.

## Conclusion

**Fig. 3:**
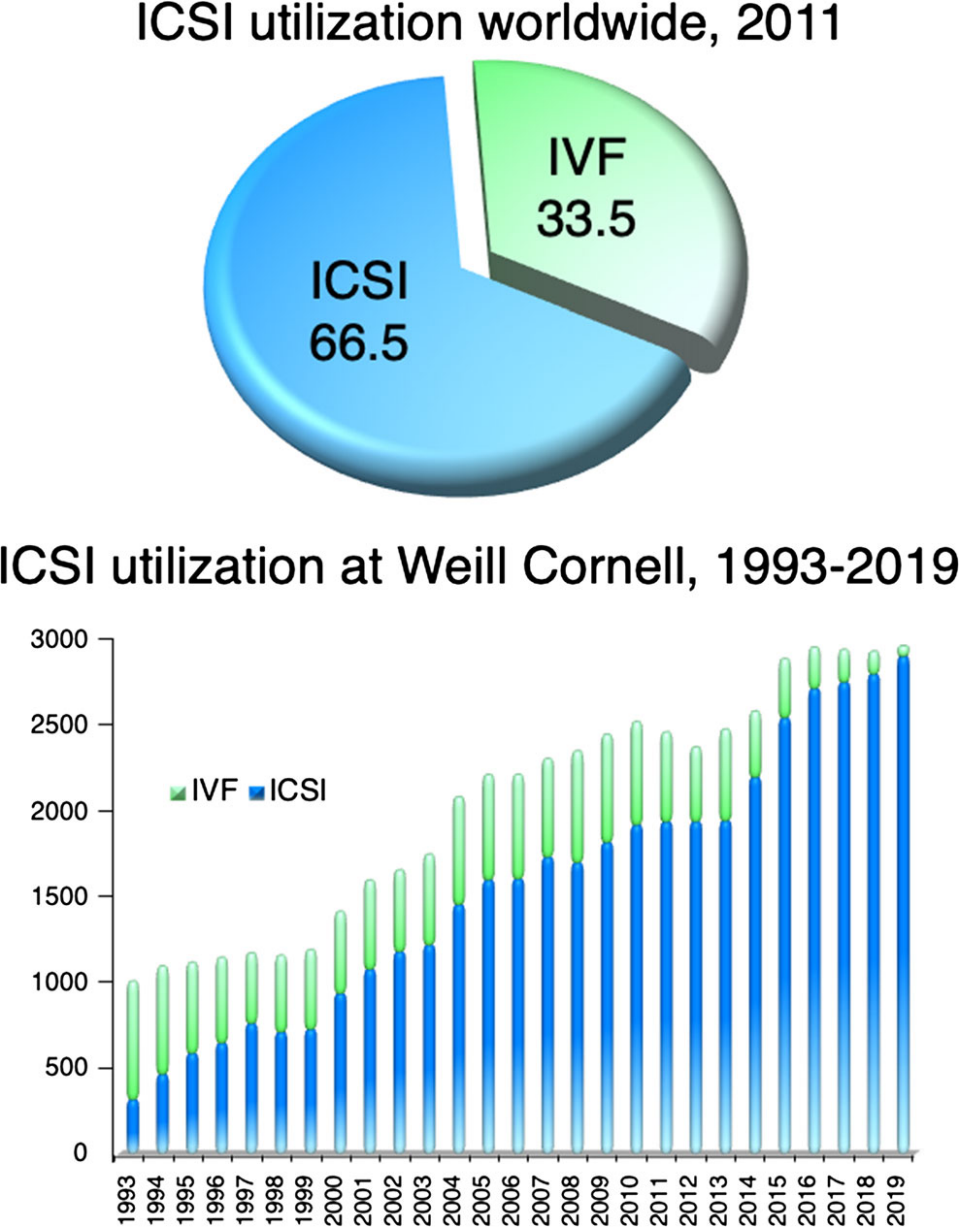
According to the most recent ICMART report, ICSI utilization was 66.5% worldwide in 2011 [1]. At Weill Cornell, our ICSI utilization has steadily increased from 1993 to 2019, with ICSI now utilized in over 95% of ART cycles done at the center

Intracytoplasmic sperm injection has become an indispensable procedure in every ART center, and its implementation has given rise to as much controversy as it has awe (Fig. 3). Indeed, every aspect of this microinvasive technique has been investigated, from different approaches of handling human oocytes and timing of ICSI, to current male factor indications and emerging non-male factor indications, to possible future roles in hereditary genome editing and neogametogenesis. As in many areas of practical laboratory ARTs, for ICSI there is no one clear and uniform utilization protocol being used worldwide. It seems clear that a male factor indication for ICSI utilization is unwavering while debate continues regarding non-male factor indications. Some ART procedures, such as following oocyte cryopreservation, remain as a popular indication ICSI utilization with preferences are mostly related to individual laboratory practice patterns. As such they portray ICSI as some sort of evolution of the in vitro fertilization process itself. In conclusion, we are left to wonder whether it is more important to identify the real indications of ICSI, or to accept ICSI as a technique offering more consistent and versatile outcomes we have come to expect from our embryology laboratories.

## References

[1] Adamson G, de Mouzon J, Chambers G, Zegers-Hochschild F, Mansour R, Ishihara O (2018). International Committee for Monitoring Assisted Reproductive Technology: world report on assisted reproductive technology, 2011. Fertil Steril.

[2] SART. Final National Summary Report for 2017. 2017. Available from: https://www.sartcorsonline.com/rptCSR_PublicMultYear.aspx?reportingYear=2017. Accessed 22 July 2020.

[3] NCI. Annual Report to the Nation. 2020. Overall Cancer Statistics 2020. Available from: https://seer.cancer.gov/report_to_nation/statistics.html. Accessed 29 July 2020.

[4] Steptoe PC, Edwards RG (1976). Reimplantation of a human embryo with subsequent tubal pregnancy. Lancet (London, England).

[5] Steptoe PC, Edwards RG (1978). Birth after the reimplantation of a human embryo. Lancet (London, England).

[6] Wang J, Sauer MV (2006). In vitro fertilization (IVF): a review of 3 decades of clinical innovation and technological advancement. Ther Clin Risk Manag.

[7] Edwards RG, Steptoe PC (1983). Current status of in-vitro fertilisation and implantation of human embryos. Lancet..

[8] Asch RH, Ellsworth LR, Balmaceda JP, Wong PC (1984). Pregnancy after translaparoscopic gamete intrafallopian transfer. Lancet..

[9] Hamori M, Stuckensen JA, Rumpf D, Kniewald T, Kniewald A, Marquez MA (1988). Zygote intrafallopian transfer (ZIFT): evaluation of 42 cases. Fertil Steril.

[10] Cohen J, Malter H, Wright G, Kort H, Massey J, Mitchell D (1989). Partial zona dissection of human oocytes when failure of zona pellucida penetration is anticipated. Hum Reprod (Oxford, England).

[11] Cohen J, Edwards R, Fehilly C, Fishel S, Hewitt J, Purdy J (1985). In vitro fertilization: a treatment for male infertility. Fertil Steril.

[12] Svalander P, Wikland M, Jakobsson A-H, Forsberg A-S (1994). Subzonal insemination (SUZI) or in vitro fertilization (IVF) in microdroplets for the treatment of male-factor infertility. J Assist Reprod Genet.

[13] Palermo G, Rosenwaks Z (1995). Assisted fertilization for male-factor infertility. Semin Reprod Med.

[14] Yanagimachi R (1984). Zona-free hamster eggs: Their use in assessing fertilizing capacity and examining chromosomes of human spermatozoa. Gamete Res.

[15] Kiessling AA, Loutradis D, McShane PM, Jackson KV (1988). Fertilization in Trypsin–Treated Oocytes. Ann N Y Acad Sci.

[16] Gordon JW, Grunfeld L, Garrisi GJ, Talansky BE, Richards C, Laufer N (1988). Fertilization of human oocytes by sperm from infertile males after zona pellucida drilling. Fertil Steril.

[17] Gordon JW, Talansky BE (1986). Assisted fertilization by zona drilling: a mouse model for correction of oligospermia. J Exp Zool.

[18] Tucker MJ, Bishop FM, Cohen J, Wiker SR, Wright G (1991). Routine application of partial zona dissection for male factor infertility. Hum Reprod.

[19] Ng SC, Bongso A, Ratnam SS, Sathananthan H, Chan CL, Wong PC (1988). Pregnancy after transfer of sperm under zona. Lancet (London, England).

[20] Fishel S, Timson J, Lisi F, Rinaldi L (1992). Evaluation of 225 patients undergoing subzonal insemination for the procurement of fertilization in vitro. Fertil Steril.

[21] Palermo G, Joris H, Devroey P, Van Steirteghem AC (1992). Induction of acrosome reaction in human spermatozoa used for subzonal insemination. Hum Reprod.

[22] Yanagimachi R, Noda YD (1970). Physiological changes in the postnuclear cap region of mammalian spermatozoa: a necessary preliminary to the membrane fusion between sperm and egg cells. J Ultrastruct Res.

[23] Palermo G, Joris H, Devroey P, Van Steirteghem AC (1992). Pregnancies after intracytoplasmic injection of single spermatozoon into an oocyte. Lancet (London, England).

[24] Hiramoto Y (1962). An analysis of the mechanism of fertilization by means of enucleation of sea urchin eggs. Exp Cell Res.

[25] Hiramoto Y (1962). Microinjection of the live spermatozoa into sea urchin eggs. Exp Cell Res.

[26] Uehara T, Yanagimachi R (1977). Activation of hamster eggs by pricking. J Exp Zool.

[27] Uehara T, Yanagimachi R (1977). Behavior of nuclei of testicular, caput and cauda epididymal spermatozoa injected into hamster eggs. Biol Reprod.

[28] Iritani A, Utsumi K, Miyake M, Hosoi Y, Saeki K (1988). In vitro fertilization by a routine method and by micromanipulation. Ann N Y Acad Sci.

[29] Goto K, Kinoshita A, Takuma Y, Ogawa K (1990). Fertilisation of bovine oocytes by the injection of immobilised, killed spermatozoa. Vet Rec.

[30] Lanzendorf SE, Maloney MK, Veeck LL, Slusser J, Hodgen GD, Rosenwaks Z (1988). A preclinical evaluation of pronuclear formation by microinjection of human spermatozoa into human oocytes. Fertil Steril.

[31] Veeck L, Oehnninger S, Acosta A, Muasher S (1989). Sperm microinjection in a clinical in vitro fertilization program. Proceedings of the 45th Annual Meeting of the American Fertility Society.

[32] Palermo G, Joris H, Derde MP, Camus M, Devroey P, Van Steirteghem A (1993). Sperm characteristics and outcome of human assisted fertilization by subzonal insemination and intracytoplasmic sperm injection. Fertil Steril.

[33] Palermo GD, Cohen J, Alikani M, Adler A, Rosenwaks Z (1995). Intracytoplasmic sperm injection: a novel treatment for all forms of male factor infertility. Fertil Steril.

[34] Van Steirteghem AC, Nagy Z, Joris H, Liu J, Staessen C, Smitz J (1993). High fertilization and implantation rates after intracytoplasmic sperm injection. Hum Reprod.

[35] Hashimoto H, Ishikawa T, Goto S, Kokeguchi S, Fujisawa M, Shiotani M (2010). The effects of severity of oligozoospermia on intracytoplasmic sperm injection (ICSI) cycle outcome. Syst Biol Reprod Med.

[36] Ketabchi AA (2016). Intracytoplasmic sperm injection outcomes with freshly ejaculated sperms and testicular or epididymal sperm extraction in patients with idiopathic cryptozoospermia. Nephro-urol Mon.

[37] Rubino P, Viganò P, Luddi A, Piomboni P (2016). The ICSI procedure from past to future: a systematic review of the more controversial aspects. Hum Reprod Update.

[38] Kovacic B, Vlaisavljevic V, Reljic M (2006). Clinical use of pentoxifylline for activation of immotile testicular sperm before ICSI in patients with azoospermia. J Androl.

[39] Ebner T, Shebl O, Mayer RB, Moser M, Costamoling W, Oppelt P (2014). Healthy live birth using theophylline in a case of retrograde ejaculation and absolute asthenozoospermia. Fertil Steril.

[40] Ebner T, Tews G, Mayer RB, Ziehr S, Arzt W, Costamoling W (2011). Pharmacological stimulation of sperm motility in frozen and thawed testicular sperm using the dimethylxanthine theophylline. Fertil Steril.

[41] Nordhoff V (2015). How to select immotile but viable spermatozoa on the day of intracytoplasmic sperm injection? An embryologist’s view. Andrology..

[42] Soares JB, Glina S, Antunes N, Wonchockier R, Galuppo AG, Mizrahi FE (2003). Sperm tail flexibility test: a simple test for selecting viable spermatozoa for intracytoplasmic sperm injection from semen samples without motile spermatozoa. Rev Hosp Clin Fac Med Sao Paulo.

[43] Ramu S, Jeyendran RS (2013). The hypo-osmotic swelling test for evaluation of sperm membrane integrity. Methods Mol Biol.

[44] Sallam HN, Farrag A, Agameya AF, Ezzeldin F, Eid A, Sallam A (2001). The use of a modified hypo-osmotic swelling test for the selection of viable ejaculated and testicular immotile spermatozoa in ICSI. Hum Reprod.

[45] Dumont A, Barbotin AL, Lefebvre-Khalil V, Mitchell V, Rigot JM, Boitrelle F (2017). Necrozoospermia: from etiologic diagnosis to therapeutic management. Gynecol Obstet Fertil Senol.

[46] French DB, Sabanegh ES, Goldfarb J, Desai N (2010). Does severe teratozoospermia affect blastocyst formation, live birth rate, and other clinical outcome parameters in ICSI cycles?. Fertil Steril.

[47] Dam AH, Feenstra I, Westphal JR, Ramos L, van Golde RJ, Kremer JA (2007). Globozoospermia revisited. Hum Reprod Update.

[48] Kuentz P, Vanden Meerschaut F, Elinati E, Nasr-Esfahani MH, Gurgan T, Iqbal N (2013). Assisted oocyte activation overcomes fertilization failure in globozoospermic patients regardless of the DPY19L2 status. Hum Reprod.

[49] Tavalaee M, Nomikos M, Lai FA, Nasr-Esfahani MH (2018). Expression of sperm PLCζ and clinical outcomes of ICSI-AOA in men affected by globozoospermia due to DPY19L2 deletion. Reprod BioMed Online.

[50] Chansel-Debordeaux L, Dandieu S, Bechoua S, Jimenez C (2015). Reproductive outcome in globozoospermic men: update and prospects. Andrology..

[51] Coutton C, Escoffier J, Martinez G, Arnoult C, Ray PF (2015). Teratozoospermia: spotlight on the main genetic actors in the human. Hum Reprod Update.

[52] Cheung S, Xie P, Parrella A, Keating D, Rosenwaks Z, Palermo GD. Identification and treatment of men with phospholipase Cζ–defective spermatozoa. Fertil Steril. 2020;114(3):535–44. 10.1016/j.fertnstert.2020.04.044 32712020

[53] Ben Khelifa M, Coutton C, Zouari R, Karaouzène T, Rendu J, Bidart M (2014). Mutations in DNAH1, which encodes an inner arm heavy chain dynein, lead to male infertility from multiple morphological abnormalities of the sperm flagella. Am J Hum Genet.

[54] El Khouri E, Thomas L, Jeanson L, Bequignon E, Vallette B, Duquesnoy P (2016). Mutations in DNAJB13, encoding an HSP40 family member, cause primary ciliary dyskinesia and male infertility. Am J Hum Genet.

[55] Hattori H, Nakajo Y, Ito C, Toyama Y, Toshimori K, Kyono K (2011). Birth of a healthy infant after intracytoplasmic sperm injection using pentoxifylline-activated sperm from a patient with Kartagener’s syndrome. Fertil Steril.

[56] Pennarun G, Chapelin C, Escudier E, Bridoux AM, Dastot F, Cacheux V (2000). The human dynein intermediate chain 2 gene (DNAI2): cloning, mapping, expression pattern, and evaluation as a candidate for primary ciliary dyskinesia. Hum Genet.

[57] Olmedo SB, Nodar F, Chillik C, Chemes HE (1997). Successful intracytoplasmic sperm injection with spermatozoa from a patient with dysplasia of the fibrous sheath and chronic respiratory disease. Hum Reprod.

[58] Nijs M, Vanderzwalmen P, Vandamme B, Segal-Bertin G, Lejeune B, Segal L (1996). Fertilizing ability of immotile spermatozoa after intracytoplasmic sperm injection. Hum Reprod.

[59] Papadimas J, Tarlatzis BC, Bili H, Sotiriadis T, Koliakou K, Bontis J (1997). Therapeutic approach of immotile cilia syndrome by intracytoplasmic sperm injection: a case report. Fertil Steril.

[60] Olmedo SB, Rawe VY, Nodar FN, Galaverna GD, Acosta AA, Chemes HE (2000). Pregnancies established through intracytoplasmic sperm injection (ICSI) using spermatozoa with dysplasia of fibrous sheath. Asian J Androl.

[61] Kordus RJ, Price RL, Davis JM, Whitman-Elia GF (2008). Successful twin birth following blastocyst culture of embryos derived from the immotile ejaculated spermatozoa from a patient with primary ciliary dyskinesia: a case report. J Assist Reprod Genet.

[62] McLachlan RI, Ishikawa T, Osianlis T, Robinson P, Merriner DJ, Healy D (2012). Normal live birth after testicular sperm extraction and intracytoplasmic sperm injection in variant primary ciliary dyskinesia with completely immotile sperm and structurally abnormal sperm tails. Fertil Steril.

[63] Yildirim G, Ficicioglu C, Akcin O, Attar R, Tecellioglu N, Yencilek F (2009). Can pentoxifylline improve the sperm motion and ICSI success in the primary ciliary dyskinesia?. Arch Gynecol Obstet.

[64] Nakamura Y, Kitamura M, Nishimura K, Koga M, Kondoh N, Masami T (2001). Chromosomal variants among 1790 infertile men. Int J Urol.

[65] Samplaski MK, Lo KC, Grober ED, Millar A, Dimitromanolakis A, Jarvi KA (2014). Phenotypic differences in mosaic Klinefelter patients as compared with non-mosaic Klinefelter patients. Fertil Steril.

[66] Hawksworth DJ, Szafran AA, Jordan PW, Dobs AS, Herati AS (2018). Infertility in patients with Klinefelter syndrome: optimal timing for sperm and testicular tissue cryopreservation. Rev Urol.

[67] Selice R, Di Mambro A, Garolla A, Ficarra V, Iafrate M, Ferlin A (2010). Spermatogenesis in Klinefelter syndrome. J Endocrinol Investig.

[68] Aksglaede L, Jørgensen N, Skakkebæk NE, Juul A (2009). Low semen volume in 47 adolescents and adults with 47,XXY Klinefelter or 46,XX male syndrome. Int J Androl.

[69] Corona G, Pizzocaro A, Lanfranco F, Garolla A, Pelliccione F, Vignozzi L (2017). Sperm recovery and ICSI outcomes in Klinefelter syndrome: a systematic review and meta-analysis. Hum Reprod Update.

[70] Tournaye H, Staessen C, Liebaers I, Van Assche E, Devroey P, Bonduelle M (1996). Testicular sperm recovery in nine 47,XXY Klinefelter patients. Hum Reprod.

[71] Palermo GD, Schlegel PN, Sills ES, Veeck LL, Zaninovic N, Menendez S (1998). Births after intracytoplasmic injection of sperm obtained by testicular extraction from men with nonmosaic Klinefelter’s syndrome. N Engl J Med.

[72] Kasman AM, Del Giudice F, Eisenberg ML (2020). New insights to guide patient care: the bidirectional relationship between male infertility and male health. Fertil Steril.

[73] Zganjar A, Nangia A, Sokol R, Ryabets A, Samplaski MK (2020). Fertility in adolescents with Klinefelter syndrome: a survey of current clinical practice. J Clin Endocrinol Metab.

[74] Martínez MC, Bernabé MJ, Gómez E, Ballesteros A, Landeras J, Glover G (2000). Screening for AZF deletion in a large series of severely impaired spermatogenesis patients. J Androl.

[75] Pinho A, Barros A, Fernandes S (2020). Clinical and molecular characterization of Y microdeletions and X-linked CNV67 implications in male fertility: a 20-year experience. Andrology..

[76] Liu XG, Hu HY, Guo YH, Sun YP. Correlation between Y chromosome microdeletion and male infertility. Genet Mol Res. 2016;15(2):1–8. 10.4238/gmr.15028426 27323142

[77] Hopps C, Mielnik A, Goldstein M (2003). D; PG, Rosenwaks Z, Schlegel PN. Detection of sperm in men with Y chromosome microdeletions of the AZFa, AZFb and AZFc regions. Hum Reprod.

[78] Krausz C, Degl’Innocenti S, Nuti F, Morelli A, Felici F, Sansone M (2006). Natural transmission of USP9Y gene mutations: a new perspective on the role of AZFa genes in male fertility. Hum Mol Genet.

[79] Ramathal C, Angulo B, Sukhwani M, Cui J, Durruthy-Durruthy J, Fang F (2015). DDX3Y gene rescue of a Y chromosome AZFa deletion restores germ cell formation and transcriptional programs. Sci Rep.

[80] Luddi A, Margollicci M, Gambera L, Serafini F, Cioni M, De Leo V (2009). Spermatogenesis in a man with complete deletion of USP9Y. N Engl J Med.

[81] Choi JM, Chung P, Veeck L, Mielnik A, Palermo GD, Schlegel PN (2004). AZF microdeletions of the Y chromosome and in vitro fertilization outcome. Fertil Steril.

[82] Katagiri Y, Neri Q, Takeuchi T, Schlegel PN, Megid W, Kent-First M (2004). Y chromosome assessment and its implications for the development of ICSI children. Reprod BioMed Online.

[83] Zhu X-B, Liu Y-L, Zhang W, Ping P, Cao X-R, Liu Y (2010). Vertical transmission of the Yq AZFc microdeletion from father to son over two or three generations in infertile Han Chinese families. Asian J Androl.

[84] McQueen DB, Zhang J, Robins JC (2019). Sperm DNA fragmentation and recurrent pregnancy loss: a systematic review and meta-analysis. Fertil Steril.

[85] Coughlan C, Clarke H, Cutting R, Saxton J, Waite S, Ledger W (2015). Sperm DNA fragmentation, recurrent implantation failure and recurrent miscarriage. Asian J Androl.

[86] Palermo GD, Neri QV, Cozzubbo T, Rosenwaks Z (2014). Perspectives on the assessment of human sperm chromatin integrity. Fertil Steril.

[87] Chi H-J, Kim S-G, Kim Y-Y, Park J-Y, Yoo C-S, Park I-H (2017). ICSI significantly improved the pregnancy rate of patients with a high sperm DNA fragmentation index. Clin Exp Reprod Med.

[88] O’Neill CL, Parrella A, Keating D, Cheung S, Rosenwaks Z, Palermo GD (2018). A treatment algorithm for couples with unexplained infertility based on sperm chromatin assessment. J Assist Reprod Genet.

[89] Benchaib M, Braun V, Lornage J, Hadj S, Salle B, Lejeune H (2003). Sperm DNA fragmentation decreases the pregnancy rate in an assisted reproductive technique. Hum Reprod.

[90] Casanovas A, Ribas-Maynou J, Lara-Cerrillo S, Jimenez-Macedo AR, Hortal O, Benet J (2019). Double-stranded sperm DNA damage is a cause of delay in embryo development and can impair implantation rates. Fertil Steril.

[91] Lafuente R, González-Comadrán M, Solà I, López G, Brassesco M, Carreras R (2013). Coenzyme Q10 and male infertility: a meta-analysis. J Assist Reprod Genet.

[92] Parrella A, Keating D, Cheung S, Xie P, Stewart JD, Rosenwaks Z (2019). A treatment approach for couples with disrupted sperm DNA integrity and recurrent ART failure. J Assist Reprod Genet.

[93] Yanagida K (2004). Complete fertilization failure in ICSI. Hum Cell.

[94] Neri QV, Lee B, Rosenwaks Z, Machaca K, Palermo GD (2014). Understanding fertilization through intracytoplasmic sperm injection (ICSI). Cell Calcium.

[95] Yanagida K, Katayose H, Yazawa H, Kimura Y, Sato A, Yanagimachi H (1999). Successful fertilization and pregnancy following ICSI and electrical oocyte activation. Hum Reprod.

[96] Yanagida K, Morozumi K, Katayose H, Hayashi S, Sato A (2006). Successful pregnancy after ICSI with strontium oocyte activation in low rates of fertilization. Reprod BioMed Online.

[97] Heindryckx B, Van der Elst J, De Sutter P, Dhont M (2005). Treatment option for sperm- or oocyte-related fertilization failure: assisted oocyte activation following diagnostic heterologous ICSI. Hum Reprod (Oxford, England).

[98] Heindryckx B, De Gheselle S, Gerris J, Dhont M, De Sutter P (2008). Efficiency of assisted oocyte activation as a solution for failed intracytoplasmic sperm injection. Reprod BioMed Online.

[99] Ohl J, Partisani M, Wittemer C, Schmitt MP, Cranz C (2003). Stoll-Keller Fo, et al. Assisted reproduction techniques for HIV serodiscordant couples: 18 months of experience. Hum Reprod.

[100] Wu MY, Ho HN (2015). Cost and safety of assisted reproductive technologies for human immunodeficiency virus-1 discordant couples. World J Virol.

[101] Kim LU, Johnson MR, Barton S, Nelson MR, Sontag G, Smith JR (1999). Evaluation of sperm washing as a potential method of reducing HIV transmission in HIV-discordant couples wishing to have children. AIDS..

[102] Semprini AE, Macaluso M, Hollander L, Vucetich A, Duerr A, Mor G (2013). Safe conception for HIV-discordant couples: insemination with processed semen from the HIV-infected partner. Am J Obstet Gynecol.

[103] Savasi V, Parisi F, Oneta M, Laoreti A, Parrilla B, Duca P (2019). Effects of highly active antiretroviral therapy on semen parameters of a cohort of 770 HIV-1 infected men. PLoS ONE.

[104] Leruez-Ville M, de Almeida M, Tachet A, Dulioust E, Guibert J, Mandelbrot L (2002). Assisted reproduction in HIV-1-serodifferent couples: the need for viral validation of processed semen. AIDS..

[105] Semprini AE, Vucetich AG, Oneta M, Rezek D, Chelo E, Hall V (2001). Sperm washing and ICSI for men with HIV infection wishing for a child. Fertil Steril.

[106] Zafer M, Horvath H, Mmeje O, van der Poel S, Semprini AE, Rutherford G (2016). Effectiveness of semen washing to prevent human immunodeficiency virus (HIV) transmission and assist pregnancy in HIV-discordant couples: a systematic review and meta-analysis. Fertil Steril.

[107] Jefferys A, Siassakos D, Wardle P (2012). The management of retrograde ejaculation: a systematic review and update. Fertil Steril.

[108] Barazani Y, Stahl PJ, Nagler HM, Stember DS (2012). Management of ejaculatory disorders in infertile men. Asian J Androl.

[109] Nikolettos N, Al-Hasani S, Baukloh V, Schöpper B, Demirel LC, Baban N (1999). The outcome of intracytoplasmic sperm injection in patients with retrograde ejaculation. Hum Reprod.

[110] Yakass M, Woodward B, Otoo M, Hiadzi E. Case report: a healthy live birth following icsi with retrograde ejaculated sperm. Afr J Reprod Health. 2014;18(4):123–5. 25854100

[111] Zhao Y, Garcia J, Jarow JP, Wallach EE (2004). Successful management of infertility due to retrograde ejaculation using assisted reproductive technologies: a report of two cases. Arch Androl.

[112] Philippon M, Karsenty G, Bernuz B, Courbiere B, Brue T, Saïas-Magnan J (2015). Successful pregnancies and healthy live births using frozen-thawed sperm retrieved by a new modified Hotchkiss procedure in males with retrograde ejaculation: first case series. Basic Clin Androl.

[113] Meng X, Fan L, Wang T, Wang S, Wang Z, Liu J (2018). Electroejaculation combined with assisted reproductive technology in psychogenic anejaculation patients refractory to penile vibratory stimulation. Transl Androl Urol.

[114] Hovav Y, Shotland Y, Yaffe H, Almagor M (1996). Electroejaculation and assisted fertility in men with psychogenic anejaculation. Fertil Steril.

[115] Ibrahim E, Lynne CM, Brackett NL (2016). Male fertility following spinal cord injury: an update. Andrology..

[116] Ohl DA, Wolf LJ, Menge AC, Christman GM, Hurd WW, Ansbacher R (2001). Electroejaculation and assisted reproductive technologies in the treatment of anejaculatory infertility. Fertil Steril.

[117] Soeterik TFW, Veenboer PW, Lock TMTW (2014). Electroejaculation in psychogenic anejaculation. Fertil Steril.

[118] Hovav Y, Yaffe H, Zentner B, Dan-Goor M, Almagor M (2002). The use of ICSI with fresh and cryopreserved electroejaculates from psychogenic anejaculatory men. Hum Reprod.

[119] Palermo GD, Neri QV, Cozzubbo T, Cheung S, Pereira N, Rosenwaks Z (2016). Shedding light on the nature of seminal round cells. PLoS One.

[120] Zorn B, Vidmar G, Meden-Vrtovec H (2003). Seminal reactive oxygen species as predictors of fertilization, embryo quality and pregnancy rates after conventional in vitro fertilization and intracytoplasmic sperm injection. Int J Androl.

[121] Sandoval JS, Raburn D, Muasher S (2013). Leukocytospermia: overview of diagnosis, implications, and management of a controversial finding. Middle East Fertil Soc J.

[122] Wolff H, Politch JA, Martinez A, Haimovici F, Hill JA, Anderson DJ (1990). Leukocytospermia is associated with poor semen quality. Fertil Steril.

[123] Agarwal A, Mulgund A, Alshahrani S, Assidi M, Abuzenadah AM, Sharma R (2014). Reactive oxygen species and sperm DNA damage in infertile men presenting with low level leukocytospermia. Reprod Biol Endocrinol : RB&E.

[124] Jung JH, Kim MH, Kim J, Baik SK, Koh S-B, Park HJ (2016). Treatment of leukocytospermia in male infertility: a systematic review. World J Mens Health.

[125] Vicari E (2000). Effectiveness and limits of antimicrobial treatment on seminal leukocyte concentration and related reactive oxygen species production in patients with male accessory gland infection. Hum Reprod.

[126] Cavagna M, Oliveira JBA, Petersen CG, Mauri AL, Silva LFI, Massaro FC (2012). The influence of leukocytospermia on the outcomes of assisted reproductive technology. Reprod Biol Endocrinol : RB&E.

[127] Ricci G, Granzotto M, Luppi S, Giolo E, Martinelli M, Zito G (2015). Effect of seminal leukocytes on in vitro fertilization and intracytoplasmic sperm injection outcomes. Fertil Steril.

[128] Cui D, Han G, Shang Y, Liu C, Xia L, Li L (2015). Antisperm antibodies in infertile men and their effect on semen parameters: a systematic review and meta-analysis. Clin Chim Acta.

[129] J-HA ADEGHE (1993). Male subfertility due to sperm antibodies: a clinical overview. Obstet Gynecol Surv.

[130] Ayvaliotis B, Bronson R, Rosenfeld D, Cooper G (1985). Conception rates in couples where autoimmunity to sperm is detected. Fertil Steril.

[131] Bates CA (1997). Antisperm antibodies and male subfertility. Br J Urol.

[132] Kutteh WH (1999). Antisperm antibodies: do antisperm antibodies bound to spermatozoa alter normal reproductive function?. Hum Reprod.

[133] Barbonetti A, Castellini C, D’Andrea S, Cordeschi G, Santucci R, Francavilla S (2019). Prevalence of anti-sperm antibodies and relationship of degree of sperm auto-immunization to semen parameters and post-coital test outcome: a retrospective analysis of over 10 000 men. Hum Reprod.

[134] Brazdova A, Senechal H, Peltre G, Poncet P (2016). Immune Aspects of Female Infertility. Int J Fertil Steril.

[135] Lu S-M, Li X, Wang S-L, Yang X-L, Xu Y-Z, Huang L-L (2019). Success rates of in vitro fertilization versus intracytoplasmic sperm injection in men with serum anti-sperm antibodies: a consecutive cohort study. Asian J Androl.

[136] Nikolaeva MA, Krutskikh AY, Korotkova IV, Golubeva EL, Kulakov VI, Sukhikh GT (2001). Antisperm antibodies and sterility: insoluble problem or perspective trend of research?. Bull Exp Biol Med.

[137] Al-dujaily SS, Chakir WK, Hantoosh SF. Direct antisperm antibody examination of infertile men. J Med Res. 2012;12(1):36–42.

[138] Garcia PC, Rubio EM, Pereira OCM (2007). Antisperm antibodies in infertile men and their correlation with seminal parameters. Reprod Med Biol.

[139] Zini A, Phillips S, Lefebvre J, Baazeem A, Bissonnette F, Kadoch IJ (2010). Anti-sperm antibodies are not associated with sperm DNA damage: a prospective study of infertile men. J Reprod Immunol.

[140] Katsoff D, Check JH, Bollendorf A, Benfer K (1995). Chymotrypsin-galactose treatment of sperm with antisperm antibodies results in improved pregnancy rates following in vitro fertilization. Am J Reprod Immunol.

[141] Check ML, Check JH, Katsoff D, Summers-Chase D (2000). ICSI as an effective therapy for male factor with antisperm antibodies. Arch Androl.

[142] Esteves SC, Schneider DT, Verza S (2007). Influence of antisperm antibodies in the semen on intracytoplasmic sperm injection outcome. Int Braz J Urol.

[143] Gudeloglu A, Parekattil SJ (2013). Update in the evaluation of the azoospermic male. Clinics (Sao Paulo).

[144] Wosnitzer MS, Goldstein M (2014). Obstructive azoospermia. Urol Clin N Am.

[145] Urology PCotASfRMicwSfMra (2008). The management of infertility due to obstructive azoospermia. Fertil Steril.

[146] Yu J, Chen Z, Ni Y, Li Z (2012). CFTR mutations in men with congenital bilateral absence of the vas deferens (CBAVD): a systemic review and meta-analysis. Hum Reprod.

[147] Ferlin A, Raicu F, Gatta V, Zuccarello D, Palka G, Foresta C (2007). Male infertility: role of genetic background. Reprod BioMed Online.

[148] Wood S, Vang E, Troup S, Kingsland CR, Lewis-Jones DI (2002). Surgical sperm retrieval after previous vasectomy and failed reversal: clinical implications for in vitro fertilization. BJU Int.

[149] Jarow JP, Espeland MA, Lipshultz LI (1989). Evaluation of the azoospermic patient. J Urol.

[150] Marquette CM, Koonin LM, Antarsh L, Gargiullo PM, Smith JC (1995). Vasectomy in the United States, 1991. Am J Public Health.

[151] Costabile RA, Spevak M (2001). Characterization of patients presenting with male factor infertility in an equal access, no cost medical system. Urology..

[152] Schlegel PN (1999). Testicular sperm extraction: microdissection improves sperm yield with minimal tissue excision. Hum Reprod.

[153] Zini A, Sigman M (2009). Are Tests of Sperm DNA Damage Clinically Useful? Pros and Cons. J Androl.

[154] Xie P, Keating D, Parrella A, Cheung S, Rosenwaks Z, Goldstein M (2019). Sperm Genomic Integrity by TUNEL Varies Throughout the Male Genital Tract. J Urol.

[155] Shefi S, Raviv G, Eisenberg ML, Weissenberg R, Jalalian L, Levron J (2006). Posthumous sperm retrieval: analysis of time interval to harvest sperm. Hum Reprod.

[156] Van Blerkom J (1990). Occurrence and developmental consequences of aberrant cellular organization in meiotically mature human oocytes after exogenous ovarian hyperstimulation. J Electron Microsc Tech.

[157] Swain JE, Pool TB (2008). ART failure: oocyte contributions to unsuccessful fertilization. Hum Reprod Update.

[158] Reid AT, Redgrove K, Aitken RJ, Nixon B (2011). Cellular mechanisms regulating sperm-zona pellucida interaction. Asian J Androl.

[159] Avella MA, Baibakov B, Dean J (2014). A single domain of the ZP2 zona pellucida protein mediates gamete recognition in mice and humans. J Cell Biol.

[160] Huang H-L, Lv C, Zhao Y-C, Li W, He X-M, Li P (2014). Mutant ZP1 in familial infertility. N Engl J Med.

[161] Chen T, Bian Y, Liu X, Zhao S, Wu K, Yan L (2017). A recurrent missense mutation in ZP3 causes empty follicle syndrome and female infertility. Am J Hum Genet.

[162] Dai C, Hu L, Gong F, Tan Y, Cai S, Zhang S (2019). ZP2 pathogenic variants cause in vitro fertilization failure and female infertility. Genet Med.

[163] Zhao J, Zhang N-Y, Xu Z-P, Chen L-J, Zhao X, Zeng H-M (2015). Effects of abnormal zona pellucida on fertilization and pregnancy in IVF/ICSI-ET. J Reprod Contracept.

[164] Shi S-L, Yao G-D, Jin H-X, Song W-Y (2016). Zhang F-LZ, Yang H-Y, et al. Correlation between morphological abnormalities in the human oocyte zona pellucida, fertilization failure and embryonic development. Int J Clin Exp Med.

[165] Moser M, Ebner T, Sommergruber M, Gaisswinkler U, Jesacher K, Puchner M (2004). Laser-assisted zona pellucida thinning prior to routine ICSI. Hum Reprod.

[166] Choi KH, Lee JH, Yang YH, Yoon TK, Lee DR, Lee WS (2011). Efficiency of laser-assisted intracytoplasmic sperm injection in a human assisted reproductive techniques program. Clin Exp Reprod Med.

[167] Ueno S, Bodri D, Uchiyama K, Okimura T, Okuno T, Kobayashi T (2014). Developmental potential of zona pellucida-free oocytes obtained following mild in vitro fertilization. Fertil Steril.

[168] Vajta G, Rienzi L, Bavister BD (2010). Zona-free embryo culture: is it a viable option to improve pregnancy rates?. Reprod BioMed Online.

[169] Lagutina I, Lazzari G, Duchi R, Turini P, Tessaro I, Brunetti D (2007). Comparative aspects of somatic cell nuclear transfer with conventional and zona-free method in cattle, horse, pig and sheep. Theriogenology..

[170] Oback B, Wiersema AT, Gaynor P, Laible G, Tucker FC, Oliver JE (2003). Cloned cattle derived from a novel zona-free embryo reconstruction system. Cloning Stem Cells.

[171] Bianchi E, Wright GJ (2014). Izumo meets Juno: preventing polyspermy in fertilization. Cell Cycle.

[172] Gupta SK (2014). Unraveling the intricacies of mammalian fertilization. Asian J Androl.

[173] O’Neill C, Cheung S, Parrella A, Keating D, Xie P, Rosenwaks Z, Malvasi A, Baldini D (2018). Relevance of oocyte morphology on ICSI outcomes. Pick up and oocyte management.

[174] Alikani M, Palermo G, Adler A, Bertoli M, Blake M, Cohen J (1995). Intracytoplasmic sperm injection in dysmorphic human oocytes. Zygote..

[175] Serhal PF, Ranieri DM, Kinis A, Marchant S, Davies M, Khadum IM (1997). Oocyte morphology predicts outcome of intracytoplasmic sperm injection. Hum Reprod.

[176] Balakier H, Bouman D, Sojecki A, Librach C, Squire JA (2002). Morphological and cytogenetic analysis of human giant oocytes and giant embryos. Hum Reprod.

[177] Balaban B, Urman B (2006). Effect of oocyte morphology on embryo development and implantation. Reprod BioMed Online.

[178] Setti AS, Figueira RCS, Braga DPAF, Colturato SS, Iaconelli A, Borges E (2011). Relationship between oocyte abnormal morphology and intracytoplasmic sperm injection outcomes: a meta-analysis. Eur J Obstet Gynecol Reprod Biol.

[179] Merviel P, Cabry R, Chardon K, Haraux E, Scheffler F, Mansouri NB (2017). Impact of oocytes with CLCG on ICSI outcomes and their potential relation to pesticide exposure. J Ovarian Res.

[180] Yi X-F, Xi H-L, Zhang S-L, Yang J (2019). Relationship between the positions of cytoplasmic granulation and the oocytes developmental potential in human. Sci Rep.

[181] Irez T, Ocal P, Guralp O, Cetin M, Aydogan B, Sahmay S (2011). Different serum anti-Müllerian hormone concentrations are associated with oocyte quality, embryo development parameters and IVF-ICSI outcomes. Arch Gynecol Obstet.

[182] Kahraman S, Yakin K, Donmez E, Samli H, Bahce M, Cengiz G (2000). Relationship between granular cytoplasm of oocytes and pregnancy outcome following intracytoplasmic sperm injection. Hum Reprod.

[183] Otsuki J, Nagai Y, Chiba K (2007). Lipofuscin bodies in human oocytes as an indicator of oocyte quality. J Assist Reprod Genet.

[184] Chen C (1986). Pregnancy after human oocyte cryopreservation. Lancet (London, England).

[185] Porcu E, Fabbri R, Seracchioli R, Ciotti PM, Magrini O, Flamigni C (1997). Birth of a healthy female after intracytoplasmic sperm injection of cryopreserved human oocytes. Fertil Steril.

[186] Kazem R, Thompson LA, Srikantharajah A, Laing MA, Hamilton MP, Templeton A (1995). Cryopreservation of human oocytes and fertilization by two techniques: in-vitro fertilization and intracytoplasmic sperm injection. Hum Reprod.

[187] Fabbri R, Porcu E, Marsella T, Primavera MR, Seracchioli R, Ciotti PM (1998). Oocyte cryopreservation. Hum Reprod.

[188] Fabbri R, Porcu E, Marsella T, Rocchetta G, Venturoli S, Flamigni C (2001). Human oocyte cryopreservation: new perspectives regarding oocyte survival. Hum Reprod.

[189] Antinori M, Licata E, Dani G, Cerusico F, Versaci C, Antinori S (2007). Cryotop vitrification of human oocytes results in high survival rate and healthy deliveries. Reprod BioMed Online.

[190] Rienzi L, Romano S, Albricci L, Maggiulli R, Capalbo A, Baroni E (2010). Embryo development of fresh ‘versus’ vitrified metaphase II oocytes after ICSI: a prospective randomized sibling-oocyte study. Hum Reprod (Oxford, England).

[191] Smith GD, Serafini PC, Fioravanti J, Yadid I, Coslovsky M, Hassun P (2010). Prospective randomized comparison of human oocyte cryopreservation with slow-rate freezing or vitrification. Fertil Steril.

[192] Schoolcraft WB, Keller JL, Schlenker T (2009). Excellent embryo quality obtained from vitrified oocytes. Reprod BioMed Online.

[193] Nagy ZP, Anderson RE, Feinberg EC, Hayward B, Mahony MC (2017). The Human Oocyte Preservation Experience (HOPE) Registry: evaluation of cryopreservation techniques and oocyte source on outcomes. Reprod Biol Endocrinol.

[194] Ubaldi F, Anniballo R, Romano S, Baroni E, Albricci L, Colamaria S (2010). Cumulative ongoing pregnancy rate achieved with oocyte vitrification and cleavage stage transfer without embryo selection in a standard infertility program. Hum Reprod (Oxford, England).

[195] Cao Y-X, Xing Q, Li L, Cong L, Zhang Z-G, Wei Z-L (2009). Comparison of survival and embryonic development in human oocytes cryopreserved by slow-freezing and vitrification. Fertil Steril.

[196] Kuwayama M, Vajta G, Kato O, Leibo SP (2005). Highly efficient vitrification method for cryopreservation of human oocytes. Reprod BioMed Online.

[197] Diaz DG, Rodriguez-Karl MC, Moody JE, Anh-Tuan HL (2007). Survival, fertilization, and cleavage rate of frozen:thawed oocytes using a new modified slow-freeze protocol-preliminary results. Fertil Steril.

[198] Doyle JO, Richter KS, Lim J, Stillman RJ, Graham JR, Tucker MJ (2016). Successful elective and medically indicated oocyte vitrification and warming for autologous in vitro fertilization, with predicted birth probabilities for fertility preservation according to number of cryopreserved oocytes and age at retrieval. Fertil Steril.

[199] Cobo A, Diaz C (2011). Clinical application of oocyte vitrification: a systematic review and meta-analysis of randomized controlled trials. Fertil Steril.

[200] Goldman RH, Racowsky C, Farland LV, Munné S, Ribustello L, Fox JH (2017). Predicting the likelihood of live birth for elective oocyte cryopreservation: a counseling tool for physicians and patients. Hum Reprod.

[201] Massarotti C, Scaruffi P, Lambertini M, Remorgida V, Del Mastro L, Anserini P (2017). State of the art on oocyte cryopreservation in female cancer patients: a critical review of the literature. Cancer Treat Rev.

[202] Yang D, Brown SE, Nguyen K, Reddy V, Brubaker C, Winslow KL (2007). Live birth after the transfer of human embryos developed from cryopreserved oocytes harvested before cancer treatment. Fertil Steril.

[203] Martinez M, Rabadan S, Domingo J, Cobo A, Pellicer A, Garcia-Velasco JA (2014). Obstetric outcome after oocyte vitrification and warming for fertility preservation in women with cancer. Reprod BioMed Online.

[204] Specchia C, Baggiani A, Immediata V, Ronchetti C, Cesana A, Smeraldi A (2019). Oocyte cryopreservation in oncological patients: eighteen years experience of a tertiary care referral center. Front Endocrinol (Lausanne).

[205] Domingo J, Guillén V, Ayllón Y, Martínez M, Muñoz E, Pellicer A (2012). Ovarian response to controlled ovarian hyperstimulation in cancer patients is diminished even before oncological treatment. Fertil Steril.

[206] Garcia-Velasco JA, Domingo J, Cobo A, Martínez M, Carmona L, Pellicer A (2013). Five years’ experience using oocyte vitrification to preserve fertility for medical and nonmedical indications. Fertil Steril.

[207] Practice Committees of the American Society for Reproductive M, Society for Assisted Reproductive T (2012). Intracytoplasmic sperm injection (ICSI) for non-male factor infertility: a committee opinion. Fertil Steril.

[208] Veeck LL, Wortham JWE, Witmyer J, Sandow BA, Acosta AA, Garcia JE (1983). Maturation and fertilization of morphologically immature human oocytes in a program of in vitro fertilization. Fertil Steril.

[209] Parrella A, Irani M, Keating D, Chow S, Rosenwaks Z, Palermo GD (2019). High proportion of immature oocytes in a cohort reduces fertilization, embryo development, pregnancy and live birth rates following ICSI. Reprod BioMed Online.

[210] Roest J, Van Heusden AM, Zeilmaker GH, Verhoeff A (1998). Treatment policy after poor fertilization in the first IVF cycle. J Assist Reprod Genet.

[211] van der Westerlaken L, Helmerhorst F, Dieben S, Naaktgeboren N (2005). Intracytoplasmic sperm injection as a treatment for unexplained total fertilization failure or low fertilization after conventional in vitro fertilization. Fertil Steril.

[212] Holubcova Z, Kyjovska D, Martonova M, Paralova D, Klenkova T, Otevrel P (2019). Egg maturity assessment prior to ICSI prevents premature fertilization of late-maturing oocytes. J Assist Reprod Genet.

[213] Pereira N, Neri QV, Lekovich JP, Palermo GD, Rosenwaks Z (2016). The role of in-vivo and in-vitro maturation time on ooplasmic dysmaturity. Reprod BioMed Online.

[214] Walls M, Junk S, Ryan JP, Hart R (2012). IVF versus ICSI for the fertilization of in-vitro matured human oocytes. Reprod BioMed Online.

[215] Dahan MH, Tan SL, Chung J, Son WY (2016). Clinical definition paper on in vitro maturation of human oocytes. Hum Reprod.

[216] Hatirnaz S, Ata B, Hatirnaz ES, Dahan MH, Tannus S, Tan J (2018). Oocyte in vitro maturation: a sytematic review. Turk J Obstet Gynecol.

[217] Bos-Mikich A, Ferreira M, Höher M, Frantz G, Oliveira N, Dutra CG (2011). Fertilization outcome, embryo development and birth after unstimulated IVM. J Assist Reprod Genet.

[218] Strowitzki T (2013). In vitro Maturation (IVM) of human oocytes. Arch Gynecol Obstet.

[219] Cha KY, Lee DR, Cho JH, Yoon TK (2006). In vitro maturation of immature oocytes and IVF/ICSI in PCOS patients. J Indian Med Assoc.

[220] Hwang JL, Lin YH, Tsai YL (2000). In vitro maturation and fertilization of immature oocytes: a comparative study of fertilization techniques. J Assist Reprod Genet.

[221] Lim J-H, Chian R-C (2010). In Vitro Maturation of Human Immature Oocytes. J Reprod Stem Cell Biotechnol.

[222] Park JH, Jee BC, Kim SH (2016). Comparison of normal and abnormal fertilization of in vitro-matured human oocyte according to insemination method. J Obstet Gynaecol Res.

[223] Margalit T, Ben-Haroush A, Garor R, Kotler N, Shefer D, Krasilnikov N (2019). Morphokinetic characteristics of embryos derived from in-vitro-matured oocytes and their in-vivo-matured siblings after ovarian stimulation. Reprod BioMed Online.

[224] Xu H, Deng K, Luo Q, Chen J, Zhang X, Wang X (2016). High serum FSH is associated with brown oocyte formation and a lower pregnancy rate in human IVF practice. Cell Physiol Biochem.

[225] Lu C-L, Wang T-R, Yan L-Y, Xia X, Zhu X-H, Li R, et al. Gonadotropin-mediated dynamic alterations during bovine oocyte maturation in vitro1. Biol Reprod. 2014;91(2):44. 10.1095/biolreprod.114.117945 24943039

[226] Ali A, Sirard M-A (2002). Effect of the absence or presence of various protein supplements on further development of bovine oocytes during in vitro maturation1. Biol Reprod.

[227] Palermo G, Munne S, Cohen J (1994). The human zygote inherits its mitotic potential from the male gamete. Hum Reprod.

[228] Wentz AC, Repp JE, Maxson WS, Pittaway DE, Torbit CA (1983). The problem of polyspermy in in vitro fertilization. Fertil Steril.

[229] van der Ven HH, Al-Hasani S, Diedrich K, Hamerich U, Lehmann F, Krebs D (1985). Polyspermy in in vitro fertilization of human oocytes: frequency and possible causes. Ann N Y Acad Sci.

[230] Ho PC, Yeung WS, Chan YF (1994). So WW, Chan ST. Factors affecting the incidence of polyploidy in a human in vitro fertilization program. Int J Fertil Menopausal Stud.

[231] Palermo GD, Munné S, Colombero LT, Cohen J, Rosenwaks Z (1995). Genetics of abnormal human fertilization. Hum Reprod.

[232] Flaherty SP, Payne D, Swann NJ, Matthews CD (1995). Assessment of fertilization failure and abnormal fertilization after intracytoplasmic sperm injection (ICSI). Reprod Fertil Dev.

[233] Palermo GD, Alikani M, Bertoli M, Colombero LT, Moy F, Cohen J (1996). Oolemma characteristics in relation to survival and fertilization patterns of oocytes treated by intracytoplasmic sperm injection. Hum Reprod.

[234] Nagy ZP, Liu J, Joris G, Bocken G, Desmet B, van Ranst A (1995). The influence of the site of sperm deposition and mode of oolemma breakage at intracytoplasmic sperm injection on fertilization and embryo development rates. Hum Reprod.

[235] Li M, Zhao W, Xue X, Zhang S, Shi W, Shi J (2015). Three pro-nuclei (3PN) incidence factors and clinical outcomes: a retrospective study from the fresh embryo transfer of in vitro fertilization with donor sperm (IVF-D). Int J Clin Exp Med.

[236] Sachs AR, Politch JA, Jackson KV, Racowsky C, Hornstein MD, Ginsburg ES (2000). Factors associated with the formation of triploid zygotes after intracytoplasmic sperm injection. Fertil Steril.

[237] Rosen MP, Shen S, Dobson AT, Fujimoto VY, McCulloch CE, Cedars MI (2006). Triploidy formation after intracytoplasmic sperm injection may be a surrogate marker for implantation. Fertil Steril.

[238] Figueira RCS, Setti AS, Braga DPAF, Iaconelli A, Borges E (2011). Prognostic value of triploid zygotes on intracytoplasmic sperm injection outcomes. J Assist Reprod Genet.

[239] Trounson A, Webb J (1984). Fertilization of human oocytes following reinsemination in vitro. Fertil Steril.

[240] Nagy ZP, Joris H, Liu J, Staessen C, Devroey P, van Steirteghem AC (1993). Fertilization and early embryology: intracytoplasmic single sperm injection of 1-day-old unfertilized human oocytes. Hum Reprod.

[241] Yuzpe AA, Liu Z, Fluker MR (2000). Rescue intracytoplasmic sperm injection (ICSI)-salvaging in vitro fertilization (IVF) cycles after total or near-total fertilization failure. Fertil Steril.

[242] Morton PC, Yoder CS, Tucker MJ, Wright G, Brockman WDW, Kort HI (1997). Reinsemination by intracytoplasmic sperm injection of 1-day-old oocytes after complete conventional fertilization failure. Fertil Steril.

[243] Lundin K, Sjögren A, Hamberger L (1996). Reinsemination of one-day-old oocytes by use of intracytoplasmic sperm injection. Fertil Steril.

[244] Nagy ZP, Staessen C, Liu J, Joris H, Devroey P, Van Steirteghem AC (1995). Prospective, auto-controlled study on reinsemination of failed-fertilized oocytes by intracytoplasmic sperm injection. Fertil Steril.

[245] Nagy ZP, Rienzi LF, Ubaldi FM, Greco E, Massey JB, Kort HI (2006). Effect of reduced oocyte aging on the outcome of rescue intracytoplasmic sperm injection. Fertil Steril.

[246] Gelbaya TA, Potdar N, Jeve YB, Nardo LG (2014). Definition and epidemiology of unexplained infertility. Obstet Gynecol Surv.

[247] Ray A, Shah A, Gudi A, Homburg R (2012). Unexplained infertility: an update and review of practice. Reprod BioMed Online.

[248] Ruiz A, Remohí J, Minguez Y, Guanes PP, Simón C, Pellicer A (1997). The role of in vitro fertilization and intracytoplasmic sperm injection in couples with unexplained infertility after failed intrauterine insemination. Fertil Steril.

[249] Foong SC, Fleetham JA, O’Keane JA, Scott SG, Tough SC, Greene CA (2006). A prospective randomized trial of conventional in vitro fertilization versus intracytoplasmic sperm injection in unexplained infertility. J Assist Reprod Genet.

[250] Bhattacharya S, Hamilton MPR, Shaaban M, Khalaf Y, Seddler M, Ghobara T (2001). Conventional in-vitro fertilisation versus intracytoplasmic sperm injection for the treatment of non-male-factor infertility: a randomised controlled trial. Lancet (London, England).

[251] Hershlag A, Paine T, Kvapil G, Feng H, Napolitano B (2002). In vitro fertilization-intracytoplasmic sperm injection split: an insemination method to prevent fertilization failure. Fertil Steril.

[252] Jaroudi K, Al-Hassan S, Al-Sufayan H, Al-Mayman H, Qeba M, Coskun S (2003). Intracytoplasmic sperm injection and conventional in vitro fertilization are complementary techniques in management of unexplained infertility. J Assist Reprod Genet.

[253] Bungum L, Bungum M, Humaidan P, Andersen CY (2004). A strategy for treatment of couples with unexplained infertility who failed to conceive after intrauterine insemination. Reprod BioMed Online.

[254] Kim HH, Bundorf MK, Behr B, McCallum SW (2007). Use and outcomes of intracytoplasmic sperm injection for non–male factor infertility. Fertil Steril.

[255] Alasmari W, Edris F, Albar Z, Eskandar M, Sultan C, Alboush A (2018). Comparable reproductive outcomes of ICSI for couples with unexplained infertility and couples with male factor infertility. Middle East Fertil Soc J.

[256] Heindryckx B, Lierman S, Van der Elst J, Dhont M (2004). Chromosome number and development of artificial mouse oocytes and zygotes. Hum Reprod.

[257] Kuznyetsov V, Kuznyetsova I, Chmura M, Verlinsky Y (2007). Duplication of the sperm genome by human androgenetic embryo production: towards testing the paternal genome prior to fertilization. Reprod BioMed Online.

[258] Palermo GD, Takeuchi T, Rosenwaks Z (2002). Technical approaches to correction of oocyte aneuploidy. Hum Reprod.

[259] Wilson TJ, Lacham-Kaplan O, Gould J, Holloway A, Bertoncello I, Hertzog PJ (2007). Comparison of mice born after intracytoplasmic sperm injection with in vitro fertilization and natural mating. Mol Reprod Dev.

[260] Onuma A, Fujii W, Sugiura K, Naito K (2017). Efficient mutagenesis by CRISPR/Cas system during meiotic maturation of porcine oocytes. J Reprod Dev.

[261] Ma H, Marti-Gutierrez N, Park SW, Wu J, Lee Y, Suzuki K (2017). Correction of a pathogenic gene mutation in human embryos. Nature.

